# Redox-responsive peptide folding enables intracellular self-assembly and controlled nucleic acid release

**DOI:** 10.1016/j.mtbio.2026.103099

**Published:** 2026-04-03

**Authors:** Huilei Dong, Wei Xie, Wenjing Huang, Yuhua Fang, Mingshui Wang, Hong Han, Xia Wu, Chunhui Zhang, Junjie Deng, Dan Yuan, Junfeng Shi

**Affiliations:** a School of Pharmacy, Institute of Biomedical Innovation, Jiangxi Medical College, Nanchang University, Nanchang, 330031, China; b Hunan Provincial Key Laboratory of Animal Models and Molecular Medicine, State Key Laboratory of Chemo/Bio-Sensing and Chemometrics, School of Biomedical Sciences, Hunan University, Changsha, Hunan, 410082, China; c HuanKui Academy, Jiangxi Medical College, Nanchang University, Nanchang, 330031, China; d Department of Cardiology, the Central Hospital of Xiangtan (The Affiliated Hospital of Hunan University), Xiangtan, Hunan, 411100, China; e College of Biology, Hunan University, Changsha, Hunan, 410082, China; f Wenzhou Institute, University of Chinese Academy Sciences, Wenzhou, Zhejiang, 325000, China

**Keywords:** Peptide self-assembly, Intracellular delivery, Redox-responsive release, Nucleic acid therapeutics, Disulfide bond

## Abstract

Intracellular peptide self-assembly provides a powerful strategy for spatiotemporal control of biomolecular interactions, yet most existing systems rely on irreversible triggers, limiting dynamic regulation. Herein, we report a redox-responsive amphiphilic peptide with a strategically positioned intramolecular disulfide bond, enabling reversible switching between a disordered coil and a β-hairpin conformation. In oxidized state, the peptide efficiently complexes with nucleic acids and penetrates cells. Intracellular glutathione reduction cleaves the disulfide, inducing β-hairpin folding, which drives supramolecular self-assembly into nanofibrils and concomitantly releases nucleic acid cargo. This three-step, reduction-responsive, assembly and release (RAR) mechanism achieves efficient, spatiotemporally controlled intracellular delivery. Structural, biophysical, and imaging analyses confirm the redox-triggered conformational transition, intracellular assembly, and cargo dissociation. This reversible and programmable platform establishes a generalizable design principle for stimulus-responsive biomaterials and nucleic acid therapeutics.

## Highlights

• Redox-triggered conformational switch enables intracellular peptide self-assembly.
• Enhanced cellular uptake and stability achieved via cyclic peptide design.
• Multi-step RAR (Reduction-responsive, Assembly and Release) mechanism facilitates efficient nucleic acid release.
• Intracellular fibril formation and cargo dissociation directly visualized.

## Graphical abstract

An intramolecular disulfide bond locks the peptide in a disordered state, allowing nucleic acids binding and cell entry. Intracellular GSH reduction cleaves the bond, inducing β-hairpin folding, nanofibril self-assembly, and controlled cargo release.

**Figure:**
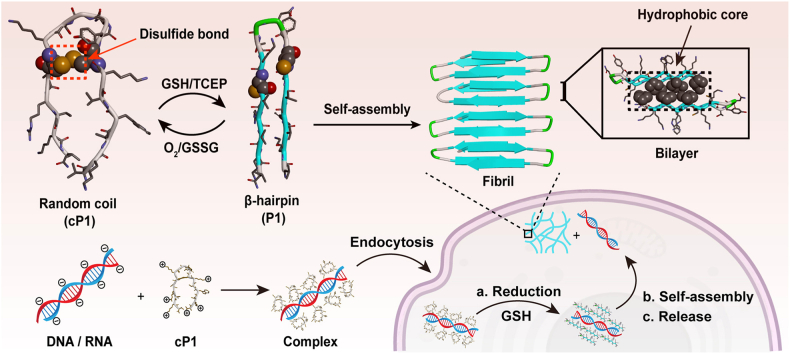

## 1 Introduction

Supramolecular assembly is a fundamental principle of life, governing processes such as cytoskeletal organization, microtubule formation, and inflammasome activation, where dynamic and reversible self-organization enables spatiotemporal regulation of cellular functions. [[1], [2], [3]]. Inspired by these natural paradigms, the construction of intracellular self-assembling systems has emerged as a powerful approach to mimic or reprogram biological events for therapeutic purposes. Peptides, with their tunable sequences, biocompatibility, and synthetic accessibility, are particularly attractive building blocks for such systems. Over the past decade, numerous intracellular triggers—including enzymes, metal ions, pH gradients, and small biomolecules—have been explored to induce peptide self-assembly. [[4], [5], [6], [7], [8], [9]]. Xu and co-workers pioneered enzyme-instructed self-assembly for applications in molecular imaging, targeted cancer therapy, and antimicrobial intervention. [8]. Schneider and colleagues demonstrated that zinc ions can drive the folding of amphiphilic β-hairpin peptides, enabling hydrogel formation [10], while Wang and co-workers exploited lysosomal acidity to induce peptide assembly for selective cancer cell killing. [11]. Moreover, bioinspired approaches have employed endogenous metabolites such as ATP to trigger nanofibril and hydrogel formation through covalent and electrostatic interactions. [12]. These examples underscore the potential of stimulus-responsive peptide systems. However, most reported strategies rely on irreversible chemical or enzymatic modifications, limiting tunability and preventing real-time, reversible control over assembly. This irreversibility is particularly limiting for applications such as intracellular delivery, where a dynamic response to the changing cellular environment is crucial for efficient cargo release and minimal off-target effects.

Disulfide bonds, one of the most conserved post-translational modifications in biology, offer an attractive mechanism for reversible, redox-sensitive regulation of protein folding and supramolecular organization. Despite their ubiquity in natural systems, their application to control synthetic peptide conformation and intracellular assembly remains largely underexplored, with most studies focusing on modulating assembly kinetics or material stability rather than actively programming conformational switches to trigger new supramolecular events. [[13], [14], [15], [16], [17]]. Intramolecular disulfide bonds, in particular, hold promise as molecular switches that can reversibly regulate peptide folding and assembly. [[18], [19], [20]]. For example, Nilsson and co-workers demonstrated that cysteine-terminally capped peptides could be locked in a non-assembling state until reductive cleavage restored their assembly competence. [20]. Nevertheless, a generalizable design strategy that exploits an intramolecular disulfide as a conformational gate to directly control peptide folding, thereby orchestrating a multi-step intracellular delivery process (membrane penetration, self-assembly, and cargo release), is still missing.

**Fig. 1:**
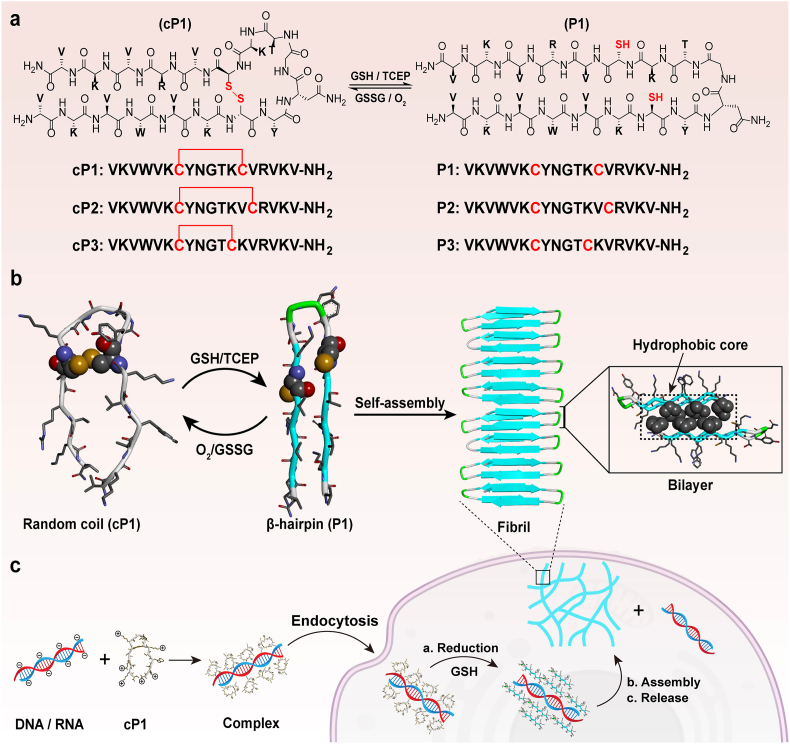
(a) Amino acid sequences of the designed peptides featuring an intramolecular disulfide bond and their reduced analogues. The disulfide linkage undergoes cleavage under reductive conditions (e.g., GSH) and can be re-formed upon oxidation (e.g., GSSG). (b) Conformational transition of **cP1** from a random coil to a β-hairpin structure (**P1**) in response to redox stimuli. **P1** further self-assembles into fibrils with a β-sheet bilayer architecture and a hydrophobic core. (c) Schematic illustration of the redox-regulated conformational switch and subsequent self-assembly process enabling intracellular nucleic acid delivery.

Herein, we report a rationally designed redox-responsive amphiphilic peptide featuring a strategically positioned intramolecular disulfide bond to regulate conformation in response to intracellular glutathione (GSH). Upon reduction, the peptide undergoes a disorder-to-β-hairpin transition, triggering supramolecular self-assembly into nanofibrils (Fig. 1a and b). The disulfide-constrained precursor exhibits enhanced membrane permeability and proteolytic stability, facilitating efficient intracellular uptake and protecting against premature degradation. Once reduced inside cells, the ensuing self-assembly induces nucleic acid dissociation and release, achieving spatiotemporally controlled intracellular delivery. This mechanism—termed **RAR** (**R**eduction-responsive, **A**ssembly, and **R**elease)—represents a dynamic, reversible, and programmable strategy for regulating intracellular peptide assembly (Fig. 1c). To the best of our knowledge, this is the first example of using conformationally gated peptide self-assembly to enhance nucleic acid delivery. Beyond drug delivery, this platform mimics nature's dynamic self-sorting processes and offers a generalizable blueprint for designing next-generation supramolecular biomaterials for diverse biomedical applications.

## 2 Materials and methods

### 2.1 Peptide synthesis

All peptides were synthesized using the standard Fmoc solid-phase peptide synthesis strategy on a CSBio synthesizer, with AM resin and coupling facilitated by DIEA and HCTU. The resin-bound peptides were cleaved and side-chain deprotected with a cocktail of TFA/phenol/water/triisopropylsilane (88:5:5:2, v/v) for 3 h under nitrogen. Afterward, the resin mixture was filtered and washed with excess TFA. The filtrate was concentrated, and the crude peptide was precipitated with cold ether. The product was purified by reverse-phase high-performance liquid chromatography (RP-HPLC) and lyophilized to yield a dry powder. All purified peptides were characterized by analytical HPLC and MALDI-TOF MS (see Figs. S23–S32 in the Supporting Information).

### 2.2 General procedure of oxidation

Briefly, reduced peptides (∼1.0 mM) were dissolved in 100 mM phosphate buffer (pH 7.4) containing two equivalents of oxidized glutathione (GSSG). The mixture was incubated at 37 °C for 2 h, and the reaction progress was monitored by analytical HPLC. The oxidized peptides were purified by RP-HPLC and lyophilized to yield dry powders.

### 2.3 Circular dichroism spectroscopy

CD spectra were recorded from 260 to 200 nm on a Jasco-815 spectrometer (Japan) under a nitrogen atmosphere. Peptides were dissolved in BTP buffer (20 mM BTP, 60 mM NaCl, pH 7.4) at 150 μM, as previously described. Samples were loaded into 1.0 mm quartz cuvettes and scanned three times at 0.5 nm intervals. Final spectra were obtained after subtracting the solvent baseline. Raw data were converted to mean residue ellipticity using the following equation, [θ] = (θ_obs_/10∗l∗c)/r, where θ_obs_ is the measured ellipticity (mdeg), l is the path length (cm), c is the concentration (M) and r is the number of residues.

### 2.4 Transmission electron microscopy

TEM images were acquired on a JEOL JEM-2100 operating at 80 kV. Briefly, 10 μL of peptide solution or diluted hydrogel was applied to a carbon-coated copper grid (200 mesh) for 1 min. Excess liquid or hydrogel was removed with filter paper, followed by three cycles of washing with water and staining with 1 wt% uranyl acetate. Grids were then air-dried before imaging.

### 2.5 Atomic force microscopy

Freshly prepared 0.5 wt% **P1** was diluted with water, and 10 μL of the solution was deposited on freshly cleaved mica for 5 min. After removing excess solution, the surface was rinsed three times with pure water blotted with filter, and dried at 37 °C.

### 2.6 Molecular modeling

The assembled conformation of **P1** was built using the structure of a β-hairpin peptide fibril (PDB ID: 2n1e) as the template, then the model was refined carefully by performing a series of energy minimization processes. Briefly, the Amber19SB force field was applied for peptide residues in vacuo using tleap module. [21]. The nonbonded cutoff for the real-space interactions was set to 12 Å. Two stages of energy minimization were conducted using a hybrid protocol of 5000 steps of steepest descent minimization followed by a conjugate gradient minimization until the convergence criterion (the root-mean-square of the energy gradient is less than 1.0 × 10^−4^ kcal/mol·Å) was satisfied or the maximum of 2000 iteration steps was reached. Then molecular dynamics simulation was used to refine the minimized conformation of the model. First, the initial structure was energy-minimized using the same approach as described above. Second, the energy-minimized structure was relaxed by performing 50 ns molecular dynamics simulation using the PMEMD module of the Amber22 software in TIP3P water with a constant temperature (T = 300 K). The SHAKE algorithm was used to restrain the covalent bonds with hydrogen atoms, and the time step for the MD simulation was set to 2 fs. [22]. The long-range electrostatic interactions were treated by using the particle mesh Ewald (PME) algorithm, and the nonbonded cutoff for the real-space interactions was set to 12 Å. [23]. The fluctuation of the backbone atoms of the residues during the simulation were calculated and analyzed. Finally, the last snapshot of the most stable model was energy-minimized again, using the same method described above, and the final conformation was used for analysis.

### 2.7 Analysis of peptide internalization mechanism

As described above, A549 cells were cultured in confocal dishes for 24 h. After discarding the culture medium, the cells were washed three times with PBS, followed by the addition of serum-free medium containing various inhibitors (CPZ: 15 μM, m- β-CD: 2.5 mM, EIPA: 30 μM) for pretreatment over 30 min [24]. One group was pretreated with medium alone and incubated at 4 °C for 30 min. The medium was then discarded, and the cells were washed once with PBS. Serum-free medium containing 10 μM FITC-labeled **cP1** and the respective inhibitors was added for a 2-h incubation. For the 4 °C pretreatment group, only serum-free medium containing 10 μM FITC-labeled peptide was added, incubated for the same duration. After incubation, the culture medium was aspirated, and the cells were washed once with medium containing 10% FBS to remove nonspecifically adsorbed peptides from the cell membrane. The cells were washed twice with PBS, stained with Hoechst 33342 nuclear dye for 15 min, and then the stain was discarded. Opti-MEM medium was added, and the inhibition of peptide entry into cells by the different inhibitors was observed under a confocal microscope.

### 2.8 Quantification of intracellular peptide

After incubating A549 cells with cP1 for 2 h, cells were counted and lysed. [23]. Liquid chromatog-raphy-mass spectrometry (LC-MS) was then performed, and the concentration of cP1 was measured using fluorescence quantitative analysis.

### 2.9 Co-localization analysis

Colocalization analysis of **cP1** and lysosomes: Serum-free medium containing 10 μM **FITC-cP1** was added, and after a 4-h incubation, the medium was removed. The cells were washed once with medium containing 10% FBS, followed by two gentle washes with PBS. LysoTracker was then added for lysosomal staining, and the cells were incubated for an additional 30 min. After removing the medium, Hoechst 33342 nuclear dye was applied for 15 min. The cells were then gently washed once with PBS, and Opti-MEM medium was added. Colocalization of **cP1** and lysosomes was observed under a confocal microscope, followed by colocalization analysis using ZEN analysis software.

Colocalization analysis of **cP1** and miRNAs: Peptides and miRNAs were pre-mixed for 30 min before being added to the cells for co-incubation over 6 h. After incubation, the culture medium was removed, and the cells were washed once with medium containing 10% FBS, followed by two gentle washes with PBS. Hoechst 33342 nuclear staining agent was then added for 15 min. The stain was removed, and Opti-MEM medium was added. Co-localization of the peptides and miRNAs was observed using a confocal microscope.

### 2.10 Fluorescence recovery after photobleaching (FRAP)

As described above, after 4 h incubation with 10 μM **FITC-cP1**, the cells were washed twice—first with medium containing 10% FBS, then with PBS. The cells were stained with LysoTracker for 30 min, washed once with PBS, and then Opti-MEM medium was added. The samples were observed using a confocal microscope.

### 2.11 Bio-TEM imaging

A549 cells (5 × 10^5^ per well) were cultured in 6-well plates, treated with 100 μM **FITC-cP1** for 8 h. After incubation, the cells were washed once with medium containing 10% FBS and then gently washed twice with ice-cold PBS. The cells were scraped using a cell scraper, centrifuged at 3000 rpm for 5 min, and the supernatant was discarded. The cells were then processed for TEM visualization through fixation, staining, dehydration, embedding, embedding, sectioning, and further staining.

### 2.12 Analysis of interaction of peptide and miRNA

Cy5-miRNA (10 μM) was pre-mixed with **FITC-cP1** stock solution (1 mM) at a charge ratio of 1:20 for 30 min. The mixture was then divided into two equal parts: one part was diluted tenfold and applied to a confocal dish, while the other part was treated with a 2.5-fold excess of TCEP (5 mM stock solution), adjusted to pH 7.4, and incubated at 37 °C for 10 min. This second mixture was also diluted tenfold and applied to a confocal dish. The distribution of miRNA and peptide were observed using a confocal microscope.

### 2.13 Particle size and zeta potential

Incubate cP1 (1 mM) with miRNA (10 μM) at a charge ratio of 20:1 at room temperature for 15 min to obtain the cP1/miRNA complex, then add deionized water to maintain the miRNA concentration at 100 nM. [25]. Measure the particle size and zeta potential of the cP1/miRNA complex using a DLS Zetasizer Nano ZS (Malvern Instruments).

## 3 Results and discussion

### 3.1 Molecular design and characterization

**Fig. 2:**
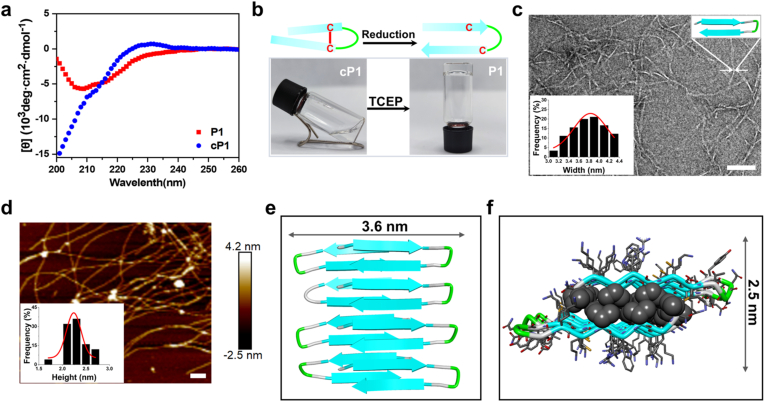
(a) CD spectra of 150 μM **cP1** and **P1** in BTP buffer (20 mM BTP, 60 mM NaCl, pH 7.4). (b) Optical images illustrating Sol-to-Gel transition of **cP1** and **P1**. The cartoon above depicts the conformational switch from random coil to β-hairpin upon reduction. (c) TEM image of 2.0 wt% hydrogel **P1**, with the inset showing the distribution of nanofiber widths (N = 90, measured from multiple fields of view across independently prepared samples). Scale bar:100 nm. (d) AFM image of 0.5 wt % gel **P1**, with the inset showing the distribution of fibril height (N = 25). A 2.0 μm × 2.0 μm area is shown. Scale bar: 200 nm. (e) Cartoon representation of the β-sheet packing, viewed perpendicular to the fibril growth axis. f) Side view of the assemblies with a Corey-Pauling-Koltun (CPK) representation of valine residues.

In our previous work, we reported the design of an amphiphilic peptide, YT-WR (VKVWVKYNGTKVRVKV-NH_2_), which adopts a β-hairpin conformation and self-assembles into a cross-β bilayer to form a nanofibril-rich hydrogel. [26]. Both our findings and those of others have established that β-hairpin conformation is a key determinant of supramolecular self-assembly and subsequently hydrogelation. [27]. This conformational dependence presents an attractive strategy to regulate peptide assembly through external stimuli such as redox signals, pH, enzymes, or metal ions. Based on this rationale, we designed a redox-responsive cyclic peptide, **cP1** (VKVWVKCYNGTKCVRVKV-NH_2_, ^7^Cys-^13^Cys, Fig. 1a) by incorporating two cysteine (C) residues at asymmetric positions within the YT-WR sequence. Here, the term “cyclic peptide” refers to a peptide conformationally constrained by an intramolecular side-chain disulfide bond, rather than a head-to-tail backbone-cyclized peptide. The intramolecular disulfide bond formed between the cysteines imposes a conformational twist, disrupting the native β-hairpin fold. Circular dichroism (CD) spectroscopy confirmed that **cP1** adopts a predominantly random coil conformation in BTP buffer, thereby preventing self-assembly and hydrogel formation (Fig. 2a). Upon reduction of the disulfide bond using tris(2-carboxyethyl) phosphine (TCEP), **cP1** is converted to its reduced form (referred to as **P1**), which adopts a non-canonical β-hairpin conformation and rapidly self-assembles into a hydrogel (Fig. 2a and b). [28,29]. Importantly, this redox-induced conformational transition is reversible, as demonstrated by repeated cycles of oxidation and reduction (Fig. S1). Oscillatory rheology further revealed the robust mechanical properties of the **P1** hydrogel. Following *in situ* gelation for 60 min, the storage modulus (G′) reached ∼3000 Pa. When subjected to 500% strain for 60 s to mimic shear-thinning, the hydrogel rapidly recovered its rigidity within seconds and achieved nearly complete recovery of G′ within 60 min (Fig. S2). This rapid self-healing behavior highlights the excellent injectability of the **P1** hydrogel and underscores its potential for biomedical applications such as tissue engineering and localized therapeutic delivery.

The secondary structure of the peptides was first characterized using circular dichroism (CD) spectroscopy to elucidate the self-assembly mechanism. As shown in Fig. S3, **P1** hydrogel exhibited a characteristic β-sheet signature with a pronounced negative peak at 216 nm, confirming the formation of β-sheets within the assemblies. Fluorescence spectroscopy further supported this finding, showing a decrease in fluorescence intensity in BTP buffer relative to that in water, along with a slight blue-shift in the emission peak of the tryptophan residues (Fig. S4). Negatively stained transmission electron microscopy (TEM) showed that **P1** formed uniform, well-defined fibrils with an average width of ≈3.8 nm, whereas **cP1** displayed no observable nanostructures under identical conditions (Fig. 2c and Fig. S5). Atomic force microscopy (AFM) imaging revealed fibrils with a height of ≈2.3 nm, consistent with the predicted thickness of a β-sheet bilayer (≈2.5 nm) (Fig. 2d and f). To gain molecular insight into the packing arrangement, we performed computational modeling of **P1** assemblies. The optimized structural model (Fig. 2e) revealed that **P1** adopts a β-hairpin conformation, forming a β-sheet bilayer with hydrophobic core. The β-hairpins in opposing layers are staggered rather than perfectly aligned, allowing valine side chains to interdigitate and stabilize the fibrillar architecture (Fig. 2f). [30]. Together, these spectroscopic, microscopic, and computational data confirm that redox-triggered β-hairpin formation drives the supramolecular assembly of **P1** into highly ordered nanofibrils.

The role of disulfide bonds in regulating peptide assembly was further examined by introducing cysteine residues at alternative positions. In **cP2**, cysteine residues were placed asymmetrically at alternative sites, resulting in a different degree of conformational disruption compared to **cP1**. Similar to **cP1**, **cP2** could be reduced to its thiol form (**P2**) under reductive conditions, triggering fibril formation (Fig. S6). CD spectroscopy confirmed that **cP2** adopts a predominantly random coil conformation in the oxidized state, whereas **P2** displays a characteristic β-hairpin signature (Fig. S7). To further test the positional effect of disulfide bonds, we designed **cP3** with symmetrically placed cysteine residues. In striking contrast to **cP1** and **cP2**, **cP3** retained its β-hairpin conformation even in the oxidized state, as confirmed by CD spectra (Fig. S8), and formed hydrogels independent of redox conditions (Fig. S9). Collectively, these results underscore that the positioning of disulfide bonds is a key determinant of conformational switching and assembly behavior.

### 3.2 Membrane permeability and mechanism

**Fig. 3:**
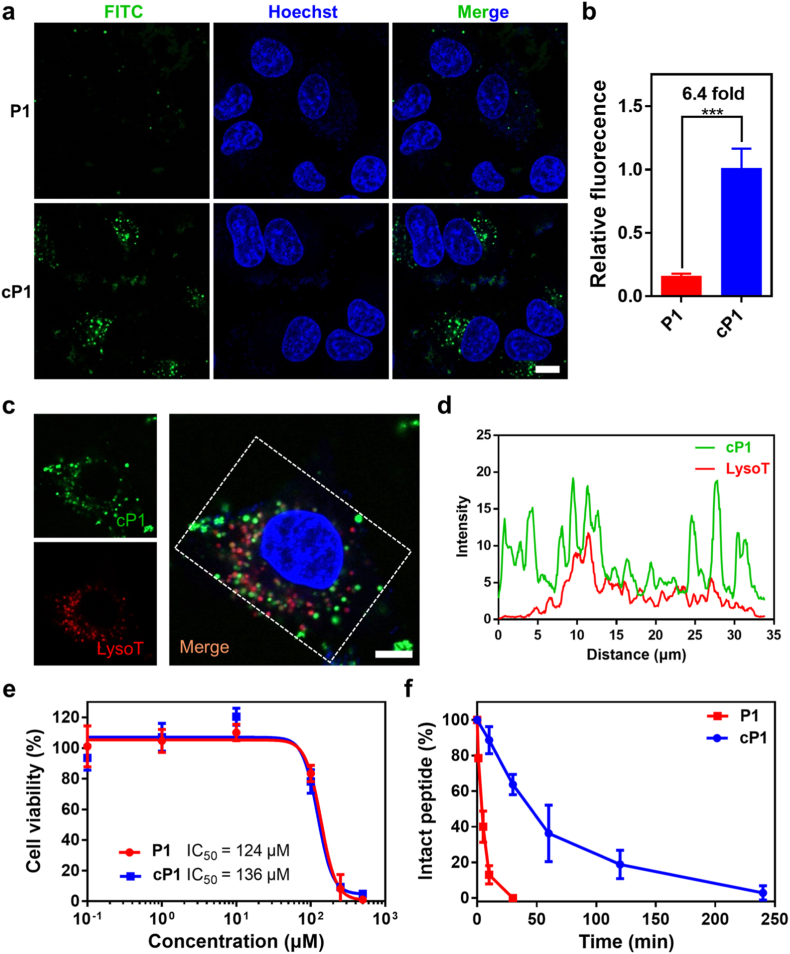
Cellular uptake, internalization mechanism, endosomal escape, cytotoxicity, and proteolytic stability of **cP1**. (a) Confocal images of A549 cells after treatment with 5 μM **FITC**-**P1** or **cP1** for 4 h. Scale bar: 10 μm. (b) Quantification of mean fluorescence intensity using ImageJ. Data are mean ± SD (n = 3). (c) Confocal images of A549 cells treated with **FITC-cP1** (green) for 4 h, co-stained with LysoTracker (red). (d) Representative colocalization profile of Fig. 3c showing endosomal escape of **cP1**. Scale bar: 10 μm. (e) Cytotoxicity of **P1** and **cP1** against SHED cells after 24 h (n = 4). (f) Proteolytic stability of **P1** and **cP1** (150 μM) upon digestion with proteinase K (0.03 U mL^−1^). Data are mean ± SD (n = 3). ∗∗∗*P* < 0.001 (applies only to panel b). (For interpretation of the references to colour in this figure legend, the reader is referred to the Web version of this article.)

Peptide amphiphiles (PA) combine hydrophobic and cationic motifs, enabling strong membrane-interacting and cell-penetrating capabilities. [31]. To evaluate the bioactivity of our redox-responsive system, we examined the cellular uptake of **cP1** and its reduced form, **P1**. Both peptides were fluorescently labeled with FITC, and A549 cells were incubated with 5 μM peptides for 4 h at 37 °C. Confocal images revealed markedly higher intracellular fluorescence for **cP1**-treated cells compared with **P1** (6.4-fold increase, Fig. 3a), as confirmed by quantitative ImageJ analysis (Fig. 3b). These findings indicate that cyclization enhances cell penetration, consistent with previous reports on cyclic peptides. [32]. Notably, **cP1** exhibited comparable uptake efficiency to penetratin, a benchmark cell-penetrating peptide (CPP), supporting its utility as a delivery scaffold (Fig. S10). A concentration-dependent increase in intracellular fluorescence further confirmed dose-responsive uptake (Fig. S11). To further evaluate the effect of disulfide bond positioning on cellular uptake, we additionally examined the internalization behavior of **cP3**, which adopts a β-hairpin conformation and remains self-assembling even in the oxidized state. Under the same conditions, **cP3** exhibited minimal intracellular fluorescence compared with **cP1**, indicating very limited cellular uptake (Fig. S12). This result suggests that disulfide bond positioning not only regulates peptide conformation and self-assembly behavior, but also strongly influences membrane permeability. The markedly lower uptake of **cP3** may be related to its constitutive self-assembling nature, which likely compromises its ability to efficiently enter cells as a delivery carrier.

Colocalization studies with LysoTracker were conducted to assess endosomal escape of **cP1** following internalization. After 4 h, colocalization between **cP1** (green) and endolysosomal marker (red) decreased substantially, indicating endosomal escape (Fig. 3c). This observation was supported by fluorescence intensity profiles from single-cell analyses (Fig. 3d). Additionally, to investigate the mechanism of internalization of **cP1**, we performed uptake studies at 4 °C and employed a panel of chemical endocytosis inhibitors: chlorpromazine (CPZ), methyl-β-cyclodextrin (m-β-CD), 5-(N-ethyl-N-isopropyl) amiloride (EIPA). [33]. Cellular uptake was almost completely abolished at 4 °C, indicating an energy-dependent mechanism (Fig. S13). CPZ had minimal effect, ruling out clathrin-mediated endocytosis as the dominant route. In contrast, uptake was significantly reduced by m-β-CD and EIPA, implicating caveolae-mediated endocytosis and macropinocytosis as the primary pathways, which was further corroborated by flow cytometry (Fig. S14).

### 3.3 Cytotoxicity and stability

The *in vitro* cytotoxicity of **cP1** and **P1** was then evaluated using SHED cells (stem cells derived from human exfoliated deciduous teeth) to establish a nontoxic working range and optimal exposure conditions for subsequent experiments. As shown in Fig. 3e, both peptides displayed comparable cytotoxicity profiles, likely attributable to their multiple positively charged lysine residues. Similar results were also observed in A549 cells (Fig. S15). Importantly, both **cP1** and **P1** maintained >80% cell viability at concentrations ≤100 μM, confirming that the concentrations used in our internalization studies (≤10 μM) are well within a nontoxic range (cytotoxicity data for **cP2** and **P2** cells are shown in Fig. S15). Because cyclic peptides are generally more resistant to enzymatic degradation than their linear counterparts [34], we next assessed the proteolytic stability of **cP1** and **P1** by subjecting them to digestion with proteinase K. As shown in Fig. 3f, linear **P1** was nearly completely degraded within 30 min, whereas ∼60% of **cP1** remained intact under identical conditions. This enhanced stability is attributed to the conformational constraint imposed by the intramolecular disulfide bond and cyclic structure. Collectively, these results indicate that **cP1** combines excellent biocompatibility with superior proteolytic resistance, suggesting it can preserve its structural integrity during systemic circulation. Together with its high cellular uptake efficiency and endosomal escape capability, these features position **cP1** as a promising and durable delivery platform for intracellular therapeutic applications.

### 3.4 Intracellular self-assembly

**Fig. 4:**
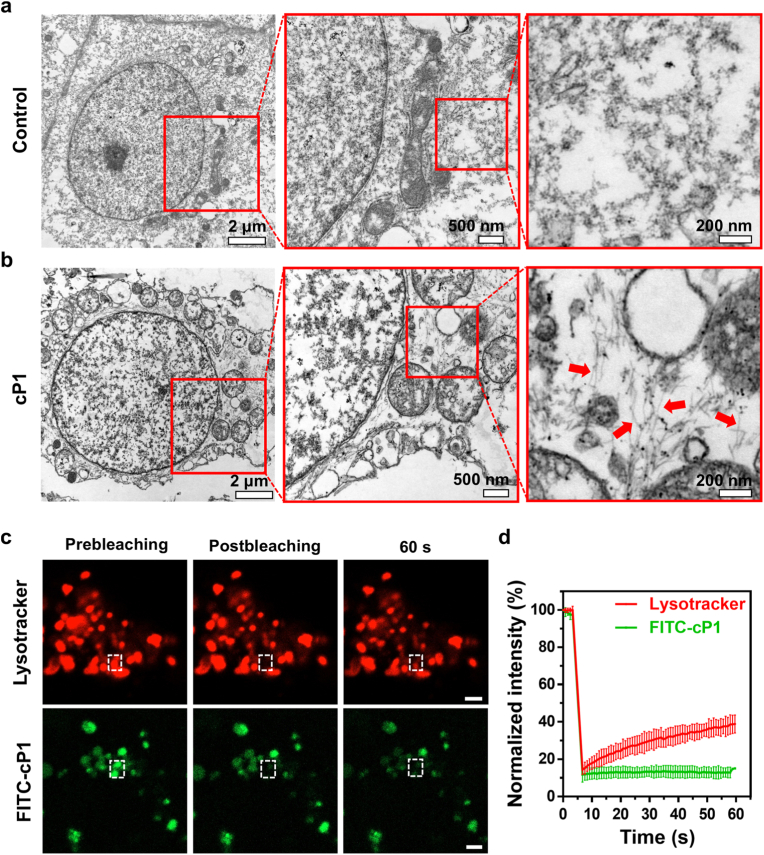
Intracellular reduction and self-assembly of **cP1**. Bio-TEM image of A549 cells in the (a) absence and (b) presence of **cP1** (100 μM, 8 h), showing extensive fibrillar networks in the cytoplasm. Red boxes indicate regions shown at higher magnification. (c) Representative FRAP images of A549 cells incubated with **FITC-cP1** (8 h) or stained with LysoTracker, acquired before and after photobleaching. (d) Quantitative FRAP recovery curves comparing LysoTracker (rapid recovery) and **FITC-cP1** (minimal recovery), confirming peptide immobilization due to intracellular self-assembly (n = 3). Scale bars: 2 μm. (For interpretation of the references to colour in this figure legend, the reader is referred to the Web version of this article.)

The intracellular milieu is characterized by a significantly elevated concentration of glutathione (GSH, 1–10 mM) compared to extracellular levels (2–20 μM), making it an attractive trigger for redox-responsive systems. [35,36]. To verify that **cP1** undergoes reduction and structural transformation within cells, A549 cells were incubated with **cP1** for 8 h, followed by cell lysis and liquid chromatography–mass spectrometry (LC-MS) analysis. As shown in Fig. S16, only reduced form (**P1**) was detected, confirming intracellular disulfide bond cleavage. To quantify intracellular accumulation, cells were lysed and analyzed fluorometrically, revealing that the intracellular concentration of **P1** reached 114 μM (Fig. S17). Consistent with this, TEM imaging of 100 μM **P1** in BTP buffer confirmed its ability to self-assemble into nanofibrils (Fig. S18). Given that **P1** exhibits self-assembly *in vitro*, we next investigated whether it assembles within the cellular environment. Bio-TEM images of **cP1**-treated cells revealed dense fibrous networks in the cytoplasm, whereas untreated control cells lacked such structures (Fig. 4a and b), suggesting intracellular self-assembly. Then, fluorescence recovery after photobleaching (FRAP) experiments were conducted to probe the molecular mobility of **FITC-cP1**. [37]. LysoTracker, a rapidly diffusing probe, was used as a control. As expected, LysoTracker fluorescence recovered fully within 60 s after photobleaching, whereas **FITC-cP1** exhibited minimal recovery, indicating peptide immobilization within supramolecular assemblies (Fig. 4c and d). [38]. Collectively, these results demonstrate that intracellular GSH reduction converts **cP1** into its self-assembling form (**P1**), driving robust nanofibril formation and establishing that redox-triggered intracellular peptide assembly occurs in living cells.

### 3.5 Intracellular nucleic acids delivery

**Fig. 5:**
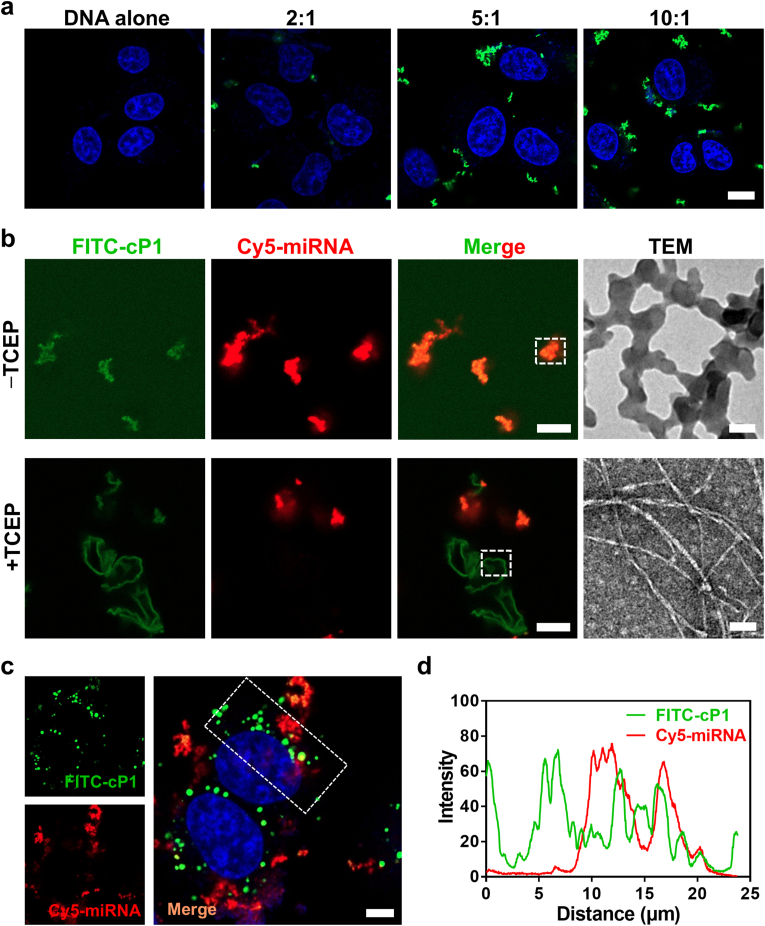
Redox-Triggered nanostructural transformation enables efficient intracellular delivery and release of nucleic acids. (a) Confocal images of A549 cells treated with complexes of peptide **cP1**/FAM-DNA at different charge ratio, showing charge-dependent internalization efficiency. Scale bar: 10 μm. (b) Confocal and TEM images of the non-covalent complex of **FITC-cP1**/Cy5-miRNA (charge ratio 20:1) before and after TCEP treatment. Scale bars: 5 μm (confocal) and 50 nm (TEM). (c) confocal images of A549 cells incubated with **cP1**/miRNA complexes for 6 h, showing intracellular distribution and release. Scale bar: 5 μm. (d) Quantitative colocalization analysis of **FITC-cP1** and Cy5-miRNA signals in Fig. 5c, confirming intracellular dissociation of the complexes.

Nucleic acid therapeutics hold great promise for treating genetic diseases and developing next-generation vaccines, yet their clinical translation remains hindered by poor cellular uptake and inefficient intracellular release. [39,40]. To assess the delivery potential of our redox-triggered self-assembling peptide system, we employed single-stranded DNA (ssDNA) as a model cargo. The cationic amphiphilic peptide cP1 efficiently complexes with anionic nucleic acids, primarily through electrostatic interactions between its positively charged residues and the negatively charged phosphate backbone of DNA/RNA, leading to the formation of nanoscale assemblies that adsorb onto the cell membrane and undergo endocytic internalization. The delivery efficiency was strongly dependent on the **cP1**/FAM-DNA (FAM labeled ssDNA) charge ratio, with higher ratios yielding enhanced uptake, likely due to stronger electrostatic interactions with the negatively charged plasma membrane (Fig. 5a). Internalization was also time-dependent, with a marked increase in intracellular fluorescence observed after 8 h of incubation (Fig. S19).

To simulate the intracellular redox environment, the reduction process was mimicked *in vitro*. Prior to reduction, colocalization analysis confirmed the formation of **cP1** (green)/Cy5-labeled miRNA (red) complexes. TEM imaging revealed nanoscale particle-like substructures, however, these features were not fully discrete and instead appeared interconnected into larger aggregate-like assemblies. Consistent with this observation, DLS measurements showed a larger hydrodynamic diameter in solution (253 nm at 30 min, increasing to 362 nm after 4 h), with a zeta potential of +0.58 ± 0.27 mV and PDI >0.5, indicating that the complexes exist as a polydisperse and dynamically evolving population under aqueous conditions (Fig. S20). Upon treatment with the reductant TCEP, the colocalization signal rapidly disappeared, and TEM revealed a morphological transition to well-defined nanofibers (Fig. 5b). This transition reflects disulfide bond cleavage, conversion of **cP1** to its β-hairpin conformation, and subsequent supramolecular self-assembly—mirroring the behavior observed for isolated **cP1** under reductive conditions (Fig. S21). These results provide a direct in-solution evidence that redox-triggered conformational switching destabilizes the peptide–nucleic acid complexes and promotes cargo dissociation. A major barrier for nucleic acid therapeutics is inefficient intracellular release, often caused by tight electrostatic binding to cationic delivery vehicles. To determine whether our redox-triggered assembly facilitated cargo release in living cells, we performed live-cell confocal microscopy. Strikingly, **cP1** and miRNA signals exhibited minimal colocalization after internalization (Fig. 5c and d), indicating successful intracellular dissociation. Then, we performed uptake studies using flow cytometry to investigate the internalization mechanism of the **cP1**/miRNA complexes. Cellular uptake was almost completely abolished at 4 °C, indicating an energy-dependent process. CPZ had minimal effect, ruling out clathrin-mediated endocytosis as the dominant route. In contrast, uptake was significantly reduced by m-β-CD and EIPA, implicating caveolae-mediated endocytosis and macropinocytosis as the primary pathways (Fig. S22). Compared with free **cP1**, the cellular uptake of the **cP1**/miRNA complexes showed a greater dependence on micropinocytosis. This difference may be associated with the nanoparticulate nature of the **cP1**/miRNA complexes, which likely influences their mode of cellular entry relative to free **cP1**. Beyond these observations, the redox-triggered *in situ* assembly constitutes a key mechanistic feature of this system. Upon intracellular reduction, **cP1** undergoes a conformational transition to a β-hairpin structure that strongly favors supramolecular self-assembly. This structural reorganization drives peptide molecules into a nanofibrillar phase, thereby reducing the pool of monomeric cationic species available to maintain electrostatic complexation with nucleic acids. Consequently, the thermodynamic equilibrium of the peptide–nucleic acid complexes shifts toward dissociation, facilitating cargo release within the cytosolic environment. In this context, *in situ* assembly is not merely a structural transformation but acts as a conformationally gated mechanism that couples redox responsiveness with supramolecular phase reorganization to regulate intracellular cargo release. Together, these results establish that the **RAR** mechanism enables not only efficient nucleic acid uptake but also controlled intracellular release, a key step toward improving the therapeutic efficacy of nucleic acid drugs.

**Fig. 6:**
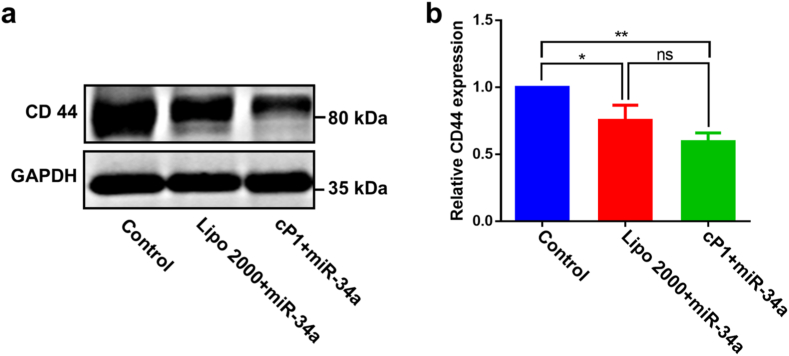
(a) Western blot analysis of CD44 expression in T24 cells treated with cP1/miRNA-34a complexes. (b) Quantification of CD44 expression using ImageJ (n = 3). ∗*P* < 0.05, ∗∗*P* < 0.01, and ns, not significance.

Our system effectively decouples the delivery and release steps: the stable, inactive cyclic form ensures safe transit and efficient uptake, whereas the triggered conformational switch and subsequent self-assembly inside the cell actively promote cargo dissociation in a controlled manner. Rather than relying solely on passive disassembly for nucleic acid release, this strategy introduces a stimulus-responsive structural mechanism to regulate intracellular release dynamics. As a proof of concept, the therapeutic potential of **cP1** for delivering miR-34a, a well-characterized tumor suppressor was evaluated. The expression of CD44, a known downstream target of miR-34a, was assessed in T24 bladder cancer cells using western blot analysis after treatment with either **cP1**/miR-34a complexes or miR-34a delivered via Lipofectamine 2000. [41]. T24 cells were selected for these studies because they exhibit high CD44 expression, making them a suitable model for assessing miR-34a–mediated gene regulation. [42]. Untreated cells served as a negative control. As shown in Fig. 6, cells treated with the **cP1**/miR-34a complexes exhibited a slightly greater reduction in CD44 expression compared to those treated with Lipofectamine 2000, highlighting the potential of **cP1** as an effective delivery vehicle for nucleic acid-based therapeutics. These results further demonstrate that cP1/miRNA complexes can efficiently enter cells and release functional miRNA into the cytoplasm. Although the detailed molecular mechanism of endosomal escape was not directly investigated in this study, the amphiphilic and cationic characteristics of **cP1** suggest a plausible contribution to membrane interactions within endosomal compartments. Similar amphiphilic cell-penetrating peptides have been reported to facilitate cytosolic delivery by inducing endosomal membrane destabilization, vesicle budding, or transient permeabilization. [43]. In particular, CPP-mediated membrane interactions have been shown to promote the release of entrapped cargo from endosomes into the cytosol. [44]. Based on these precedents, it is reasonable to speculate that **cP1** may similarly interact with endosomal membranes, thereby assisting endosomal escape of **cP1**/miRNA complexes. In addition to delivery efficiency, the biosafety of the peptide assemblies warrant consideration. After nucleic acid release, the reduced peptide **P1** remains in its self-assembled nanofibrillar state inside cells. Notably, **cP1** was designed solely as a supramolecular carrier and does not possess intrinsic biological activity. Cytotoxicity studies revealed an IC_50_ value greater than 100 μM, whereas the peptide concentration used during nucleic acid delivery (approximately 8 μM at charge ratio = 20) is substantially lower than this threshold. Moreover, the intracellular assemblies are formed through noncovalent supramolecular interactions, suggesting a dynamic rather than permanently crosslinked nature. Under the experimental conditions employed in this study, these assemblies are therefore unlikely to induce noticeable cytotoxicity.

## 4 Conclusion

In summary, we designed a redox-responsive self-assembling peptide that leverages a single disulfide bond to regulate conformation and intracellular behaviours. The disulfide-bridged cyclic peptide **cP1** demonstrates superior membrane permeability and proteolytic stability, enabling efficient and biocompatible intracellular delivery. Intracellular glutathione triggers disulfide reduction, driving spontaneous self-assembly and nanostructure formation. When applied to nucleic acid delivery, this dynamic assembly process facilitates cargo dissociation and release, overcoming a key limitation of conventional CPP-based systems. Compared with classical CPPs such as TAT and R9, **cP1** offers enhanced stability, controlled activation, and improved intracellular release efficiency. [45]. This bioinspired approach emulates natural supramolecular organization and provides a versatile platform for the rational design of smart, redox-activated delivery systems. Beyond nucleic acid delivery, the “conformationally gated self-assembly” principle established here represents a generalizable design strategy that could be readily adapted to other therapeutic cargoes (e.g., proteins, small molecules) and extracellular stimuli (e.g., enzymatic activity, changes in pH), opening new avenues for developing next-generation supramolecular biomaterials. For potential in vivo applications, the cyclic precursor of **cP1** exhibits enhanced structural stability, which may help maintain its integrity during systemic circulation prior to cellular internalization. In addition, the intracellular redox gradient provides an inherent level of selectivity, enabling activation and cargo release specifically within the cytosolic environment. Nevertheless, several challenges remain for successful in vivo translation, including systemic pharmacokinetics, biodistribution, potential off-target accumulation of peptide assemblies, and the long-term clearance of intracellular nanofibrillar structures. Future studies will therefore focus on evaluating circulation half-life, organ distribution, and in vivo therapeutic efficacy, as well as exploring structural optimization strategies such as PEGylation or the incorporation of targeting ligands to enhance tumor selectivity and reduce systemic exposure. Ultimately, these efforts will facilitate in vivo validation and broaden the applicability of this strategy to diverse nucleic acid therapeutics. [39,46,47].

## CRediT authorship contribution statement

**Huilei Dong:** Conceptualization, Data curation, Formal analysis, Funding acquisition, Investigation, Methodology, Validation, Writing – original draft. **Wei Xie:** Data curation, Formal analysis, Investigation, Methodology, Validation, Writing – review & editing. **Wenjing Huang:** Data curation, Supervision, Visualization. **Yuhua Fang:** Investigation, Methodology. **Mingshui Wang:** Investigation, Methodology. **Hong Han:** Investigation, Methodology. **Xia Wu:** Methodology, Supervision. **Chunhui Zhang:** Methodology, Software, Writing – review & editing. **Junjie Deng:** Methodology, Writing – review & editing. **Dan Yuan:** Funding acquisition, Supervision, Writing – review & editing. **Junfeng Shi:** Project administration, Resources, Supervision, Writing – review & editing.

## Declaration of competing interest

The authors declare no competing financial interests.

## Data Availability

Data will be made available on request.
