# Supplementary material for: Redox-responsive peptide folding enables intracellular self-assembly and controlled nucleic acid release
Source: Mater Today Bio. 2026 Apr 3;38:103099. doi: 10.1016/j.mtbio.2026.103099 (PMC13091059; doi:10.1016/j.mtbio.2026.103099)
Supplement: Multimedia component 1 [file mmc1.docx]

**Supporting Information**

**Redox-Responsive Peptide Folding Enables Intracellular Self-Assembly and Controlled Nucleic Acid Release**

Huilei Dong^a, b, 1^, Wei Xie^b, 1^, Wenjing Huang^b^, Yuhua Fang^c^, Mingshui Wang^b^, Hong Han^b^, Xia Wu^d^, Chunhui Zhang^e^, Junjie Deng^f, *^, Dan Yuan^b, *^, Junfeng Shi^b, *^

*^a^* *School of Pharmacy, Institute of Biomedical Innovation, Jiangxi Medical College, Nanchang University, Nanchang 330031, China.*

*^b^* *Hunan Provincial Key Laboratory of Animal Models and Molecular Medicine, State Key Laboratory of Chemo/Bio-Sensing and Chemometrics, School of Biomedical Sciences, Hunan University, Changsha, Hunan 410082, China.*

*^c^* *HuanKui Academy, Jiangxi Medical College, Nanchang University, Nanchang 330031, China.*

*^d^* *Department of Cardiology, the Central Hospital of Xiangtan (The Affiliated Hospital of Hunan University) Xiangtan, Hunan 411100, China.*

*^e^* *College of Biology, Hunan University, Changsha, Hunan 410082, China.*

*^f^* *Wenzhou Institute, University of Chinese Academy Sciences, Wenzhou, Zhejiang 325000, China.*

^1^ These authors contributed equally to this work.

^*^Corresponding authors; Email: j.deng@ucas.ac.cn, [yuandan@hnu.edu.cn](mailto:yuandan@hnu.edu.cn), [Jeff-shi@hnu.edu.cn](mailto:Jeff-shi@hnu.edu.cn)

**Table of Contents**

**Materials and Methods**

**Table S1.** Peptide sequences of all peptides studied.

**Table S2.** DNA and RNA sequences in this study.

**Figure S1.** Chromatograms showing the oxidation-reduction cycles of peptide **cP1.**

**Figure S2.** Storage (G’) and loss modulus of **P1** hydrogel under variable strain over time.

**Figure S3.** Circular dichroism (CD) spectra of a 2 wt% **P1** hydrogel.

**Figure S4.** Fluorescence emission spectra of **P1** in water and in BTP buffer.

**Figure S5.** TEM image of 2.0 wt% **cP1** solution.

**Figure S6.** Chromatograms and MS spectra of **cP2** before and after TCEP treatment, and TEM image of 2.0 wt% **cP2** solution treated with TCEP.

**Figure S7.** CD spectra of 2 wt% **P2** hydrogel and **cP2** solution.

**Figure S8.** CD spectra of 150 μM **cP3** in BTP buffer.

**Figure S9.** Optical images of **P3** hydrogel and **cP3** hydrogel.

**Figure S10.** Confocal microscopy image of cellular uptake of fluorescently labelled penetratin and quantification of mean fluorescence intensity.

**Figure S11.** Confocal images of concentration-dependent cellular internalization of FITC-labelled **cP1**.

**Figure S12.** Confocal images of A549 cells after treatment with cP3.

**Figure S13.** Confocal images showing uptake of **FITC-cP1** in the presence of endocytosis inhibitors or at 4 °C.

**Figure S14.** Quantification of **cP1** uptake using flow cytometry.

**Figure S15.** Cytotoxicity of **P1** and **cP1** against A549 cells, **P2** and **cP2** against SHED cells.

**Figure S16.** Mass spectrometry analysis of cell lysates from A549 cells treated with **cP1**.

**Figure S17.** Fluorescence quantification of cell lysates.

**Figure S18.** TEM image of 100 μM **P1** in BTP buffer.

**Figure S19.** Confocal images showing time-dependent cellular internalization of **cP1** and FAM-DNA.

**Figure S20.** DLS and zeta-potential characterization of **cP1**/miRNA complexes.

**Figure S21** TEM image of 1 mM **cP1** in BTP buffer and treated with TCEP.

**Figure S22.** Quantification of **cP1**/miRNA complexes uptake by A549 cells using flow cytometry.

**Figure S23-S32.** Analytical HPLC chromatograms and MS spectra of studied peptides.

**Materials and Methods**

**Materials**

All Fmoc-protected amino acids, Rink Amide (AM) resin, and coupling reagents were purchased from CSBio (Shanghai) ltd. Proteinase K (10 mg/mL) was purchased from Beijing Solarbio Science & Technology Co., Ltd. DL-Dithiothreitol (DTT), N,N-diisopropylethylamine (DIPEA), Fluorescein 5-isothiocyanate (FITC) and trifluoroacetic acid (TFA) were supplied by Energy Chemical (Shanghai, China). L-glutathione oxidized (GSSG) were purchased from Sigma Aldrich (Shanghai, China). Piperidine and inorganic salts (e.g., NaCl) and organic solvents (e.g., DMF, DMSO) were purchased from Sinopharm Chemical Reagent Co., Ltd. LysoTracker Red DND-99 and Hoechst 33342 were purchased from Thermo Fisher Scientific (China) Co., Ltd. Methyl- b -cyclodextrin, 5-(N-Ethyl-N-isopropyl)-Amiloride (EIPA), and chlorpromazine (CPZ) were purchased from Shanghai Topscience Co., Ltd. Fluorescently labeled DNA/RNA was synthesized by Sangon Biotechnology (Shanghai) Co., Ltd. All the solvents and reagents were used directly as received from commercial sources without further purification. CD44 and GAPDH antibodies were purchased from Proteintech Group (Wuhan, China). miRNA-34a mimics were synthesized by TsingkeBiotech Co., Ltd (Beijing, China).

**General procedure of hydrogel preparation**

In a typical experiment, 2.0 mg of peptide was dissolved in 50 μL of cold deionized water, followed by the addition of an equal volume of chilled BTP buffer (40 mM BTP, 120 mM NaCl, pH 7.4), producing a 2 wt% peptide solution. The sample was incubated at 37 °C to induce gelation, and hydrogel formation was confirmed after 1 h using the inverted tube test.

**TCEP-induced gelation**

A 2 wt% cP1 solution was prepared and incubated at 37 °C for 1 h. Subsequently, two equivalents of TCEP in BTP were added, mixed thoroughly, and the sample was returned to the 37 °C for further incubation.

**Oscillatory rheology**

The rheological properties of P1 hydrogel were evaluated using a rheometer (MCR-92, Anton Paar, Austria) equipped with a 15 mm cone-plate. In brief, a 150 μL sample was loaded between the plates with a 0.5 mm gap, and standard silicone oil was applied to prevent evaporation. Storage (G') and loss moduli (G") were measured via dynamic time sweep analysis at 37 °C, using a constant angular fre-quency of 6 rad/s and an initial strain of 0.2%. After 60 min, a 500% strain was applied for 1 minute, followed by a return to 0.2% strain for another 60 min. The process was repeated in cycles.

**Cell viability assay**

SHED cells (5000 per well) were seeded in 96-well plates with 100 μL α-MEM medium containing 15% fetal bovine serum (FBS), 100 U/mL penicillin, and 100 μg/mL streptomycin. After 24h incubation at 37°C with 5% CO_2_, the medium was replaced with fresh serum-free medium containing 0.1–500 μM peptides. Blank medium and medium without cells served as positive and negative controls, respectively. After 24 h, the medium was removed and replaced with serum-free medium containing (4,5-dimethylthiazol-2-yl)-2,5-diphenyltetrazolium bromide (MTT) at a final concentration of 0.5 mg/mL for 4 h. The medium was then replaced with 100 μL DMSO and incubating at 37°C for 30 min with shaking to dissolve formazan crystals. Absorbance was measured at 570 nm on a plate reader (Spectra Max M2). The absorbance of the negative controls was subtracted from each sample as a blank. The percent cell viability was then calculated using the formula: (Absorbance of peptide-treated cells / Absorbance of untreated cells) × 100.

**Stability assay in vitro**

Peptides (150 μM) were incubated with proteinase K (0.03 U/mL) in PBS (pH 7.4) at 37 °C. At designated time points, aliquots were quenched with 5% aqueous TFA and analyzed by analytical HPLC. Each time point was performed in triplicate.**Visualization of cellular uptake in vitro**

A549 cells (1.5 × 10⁵) were seeded in confocal dishes and cultured for 24 h. The culture medium was then replaced with serum-free medium containing FITC-labeled peptides and incubated for 4 h. Cells were washed once with medium containing 10% FBS, then twice with PBS. Nuclei were stained with Hoechst 33342 for 15 min, washed, and observed in Opti-MEM using confocal microscopy.

**Flow cytometry**

As described above, A549 cells (3 × 10⁵ per well) were cultured in 6-well plates and treated with inhibitors. After incubation, the cells were gently washed three times with PBS and digested with trypsin for 10 minutes. Digestion was halted by adding complete medium, and the cells were collected. The collected cells were gently washed twice with PBS containing 1% FBS, with both the digestion and washing processes conducted on ice. Finally, the cells were resuspended in 400 μL of PBS containing 1% FBS, filtered through a filter cloth, and analyzed. For FITC-labeled peptides, a 488 nm laser was used for excitation, and fluorescence was detected in the FITC channel.

**Quantification of intracellular peptide**

After incubating A549 cells with **cP1** for 2 h, cells were counted and lysed.[23] Liquid chromatography-mass spectrometry (LC-MS) was then performed, and the concentration of **cP1** was measured using fluorescence quantitative analysis.

**Analysis of interaction of peptide and miRNA**

Cy5-miRNA (10 μM) was pre-mixed with **FITC-cP1** stock solution (1 mM) at a charge ratio of 1:20 for 30 min. The mixture was then divided into two equal parts: one part was diluted tenfold and applied to a confocal dish, while the other part was treated with a 2.5-fold excess of TCEP (5 mM stock solution), adjusted to pH 7.4, and incubated at 37 °C for 10 min. This second mixture was also diluted tenfold and applied to a confocal dish. The distribution of miRNA and peptide were observed using a confocal microscope.

**Western blot analysis**

A miRNA-34a stock solution (10 μM) was pre-mixed with **cP1** stock solution (1 mM) at charge ratios of 1:10 for 30 min. Opti-MEM medium was then added to dilute the solution, resulting in a final miRNA-34a concentration of 100 nM. The prepared solution was added to the T24 cells and incubated for 48 h. After incubation, cells were thoroughly lysed using RIPA lysis buffer supplemented with 1 mM PMSF and centrifuged to obtain total protein. The concentration of the lysates was determined using the BCA Protein Quantification Kit (E112-01, Vazyme, Jiangsu, China). 20 μg of total protein was diluted with 5 × SDS buffer, subjected to heating for 10 minutes at 100 °C, loaded onto 10% SDS-PAGE, transferred onto PVDF membranes (Millipore, USA), and subsequently blocked with 5% skim milk in TBST. Then the membrane was probed with corresponding primary antibodies (CD44 and GAPDH). After washing, the membranes were incubated with horseradish peroxidase (HRP) conjugated secondary antibody for 1 hour at room temperature. Bands were detected by chemiluminescence (BeyoECL Plus, Beyotime, China) and imaged (JiaPeng Science Technology Co., Ltd, Shanghai, China).

**Table S1.** Peptide sequences of all peptides studied.

| Name | Sequence |
| --- | --- |
| **P1** | VKVWVKCYNGTKCVRVKV-NH_2_ |
| **P2** | VKVWVKCYNGTKVCRVKV-NH_2_ |
| **P3** | VKVWVKCYNGTCKVRVKV-NH_2_ |
| **FITC-P1** | FITC-G-VKVWVKCYNGTKCVRVKV-NH_2_ |
| **FITC-P3** | FITC-G VKVWVKCYNGTCKVRVKV-NH_2_ |

**Table S2.** DNA and RNA sequences.

| Name | Sequence |
| --- | --- |
| **FAM-DNA** | 5’-ATG TGG AAA ATC TCT AGC AGT TGG GTA GGG CGG GTT GGG ATA TAC CCA TCT GCT AGA GAT TTT-[FAM]-3’ |
| **Cy5-miRNA** | 5’-[Cy5]-UUGUACUACACAAAAGUACGU-3’ (sense) |
| **miR-34a mimics** | 5’-UGGCAGUGUCUUAGCUGGUUGU-3’ (sense) |


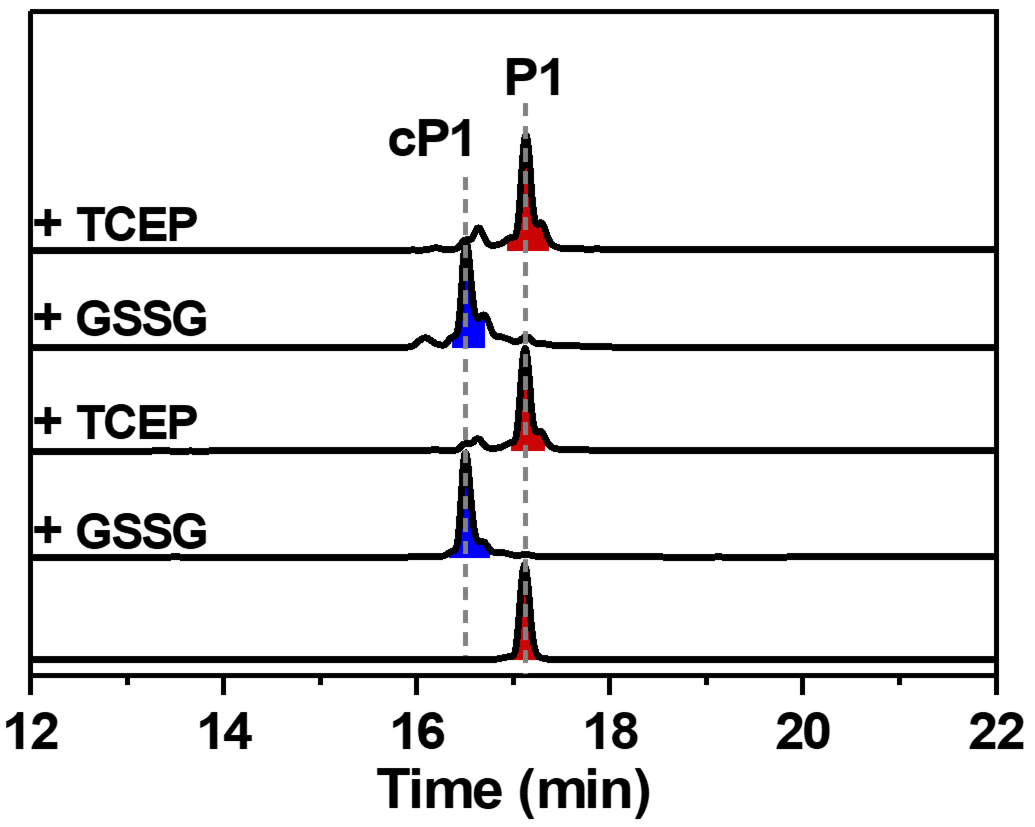


**Figure S1.** Chromatograms showing the oxidation-reduction cycles of peptide **cP1**.


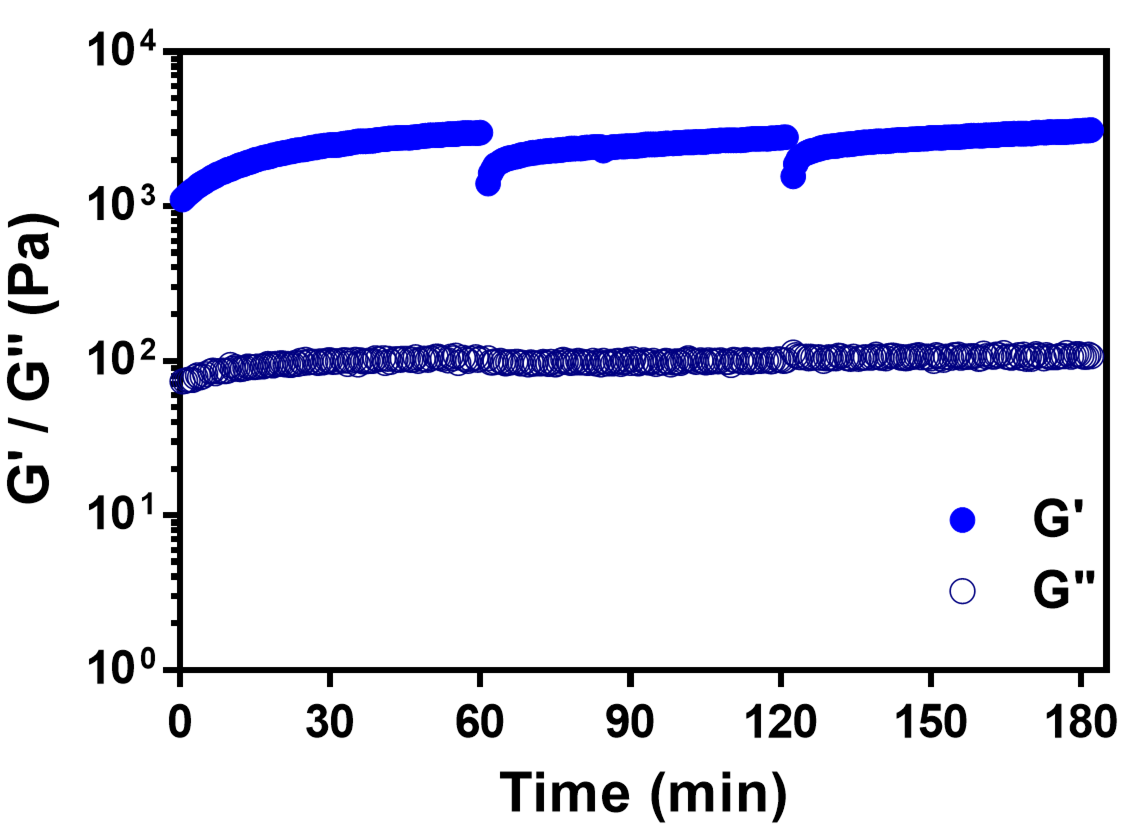


**Figure S2.** Storage (G’) and loss modulus of **P1** hydrogel under variable strain over time, demonstrating shear-thinning and recovery behavior.


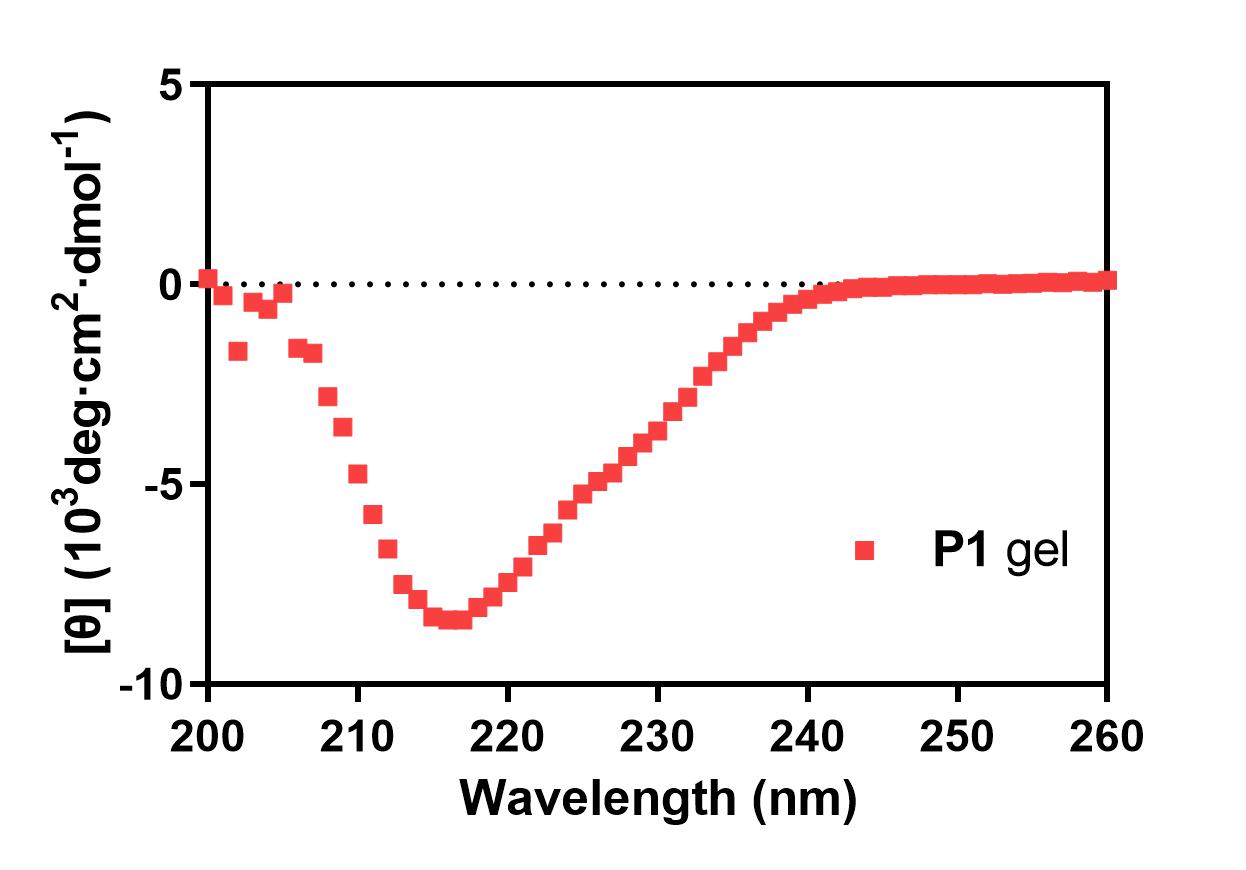


**Figure S3.** Circular dichroism (CD) spectra of a 2 wt% **P1** hydrogel.


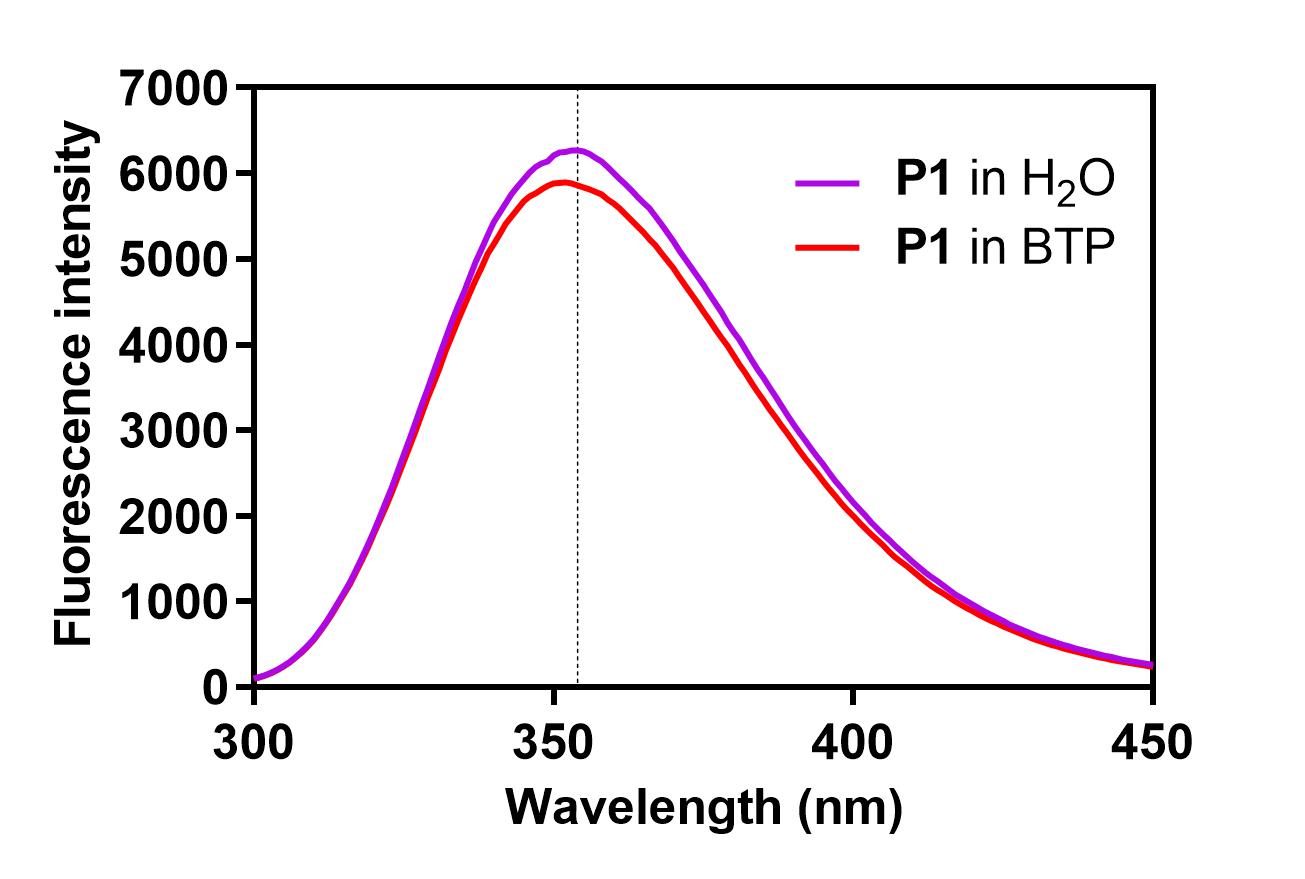


**Figure S4.** Fluorescence emission spectra of **P1** in water and in BTP buffer (20 mM BTP, 60 mM NaCl, pH 7.4).


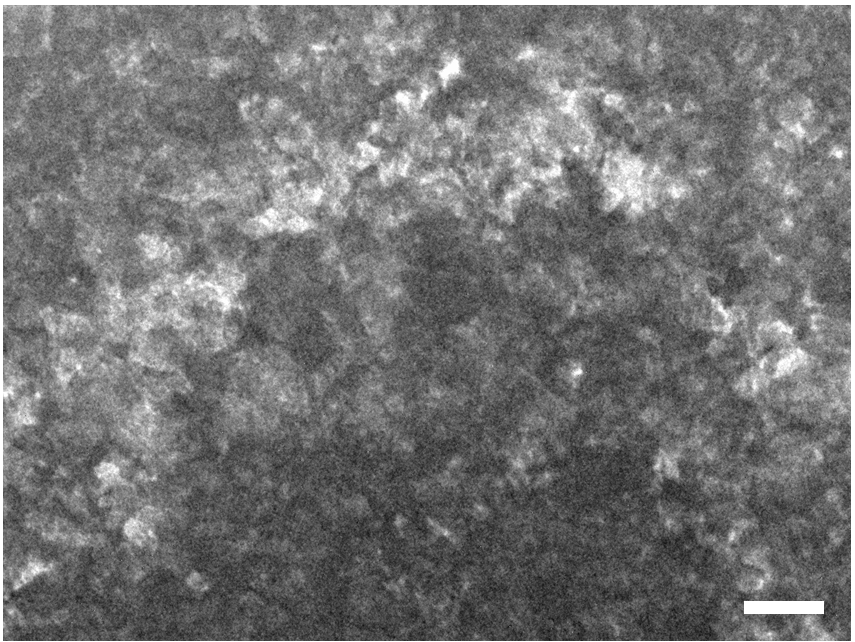


**Figure S5.** TEM image of 2.0 wt% **cP1** solution. Scale bar: 50 nm.


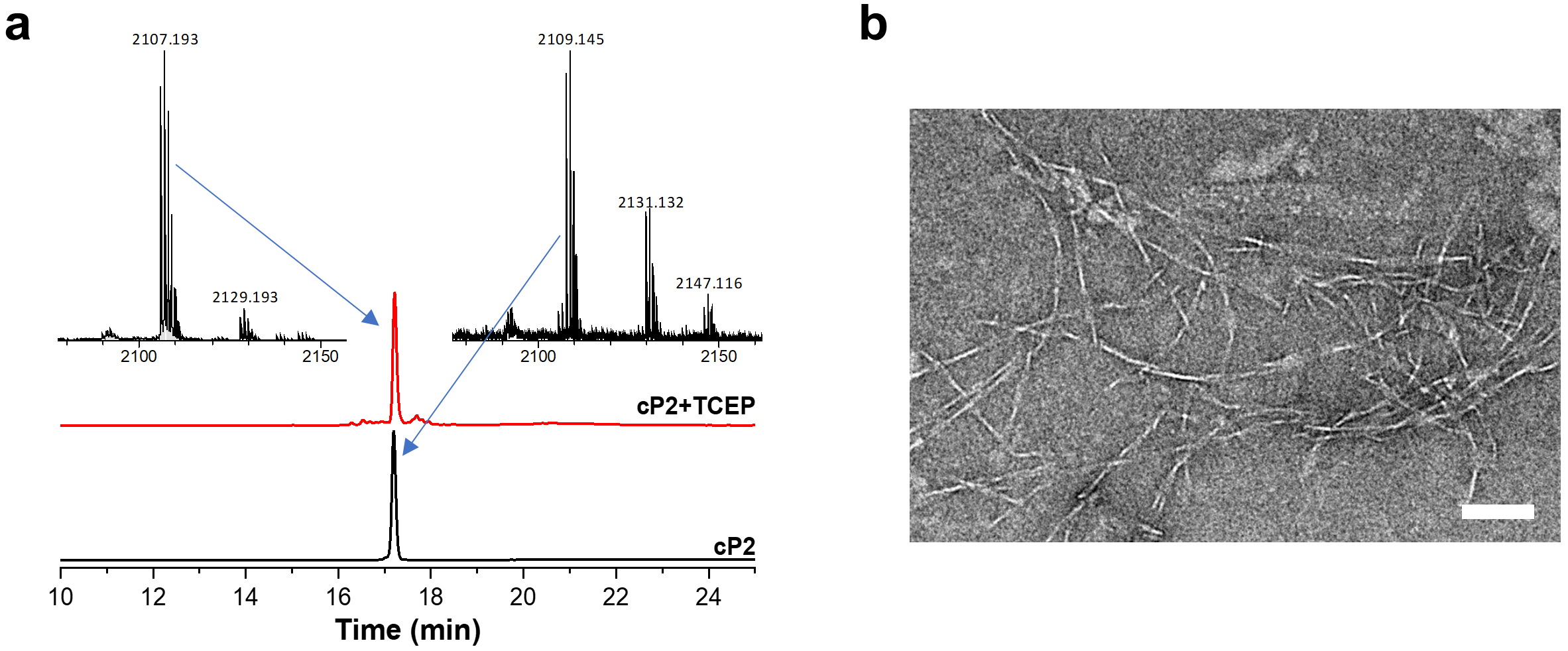


**Figure S6.** a) Chromatograms and MS spectra of **cP2** before and after TCEP treatment. b) TEM image of 2.0 wt% **cP2** solution treated with TCEP. Scale bar: 100 nm.


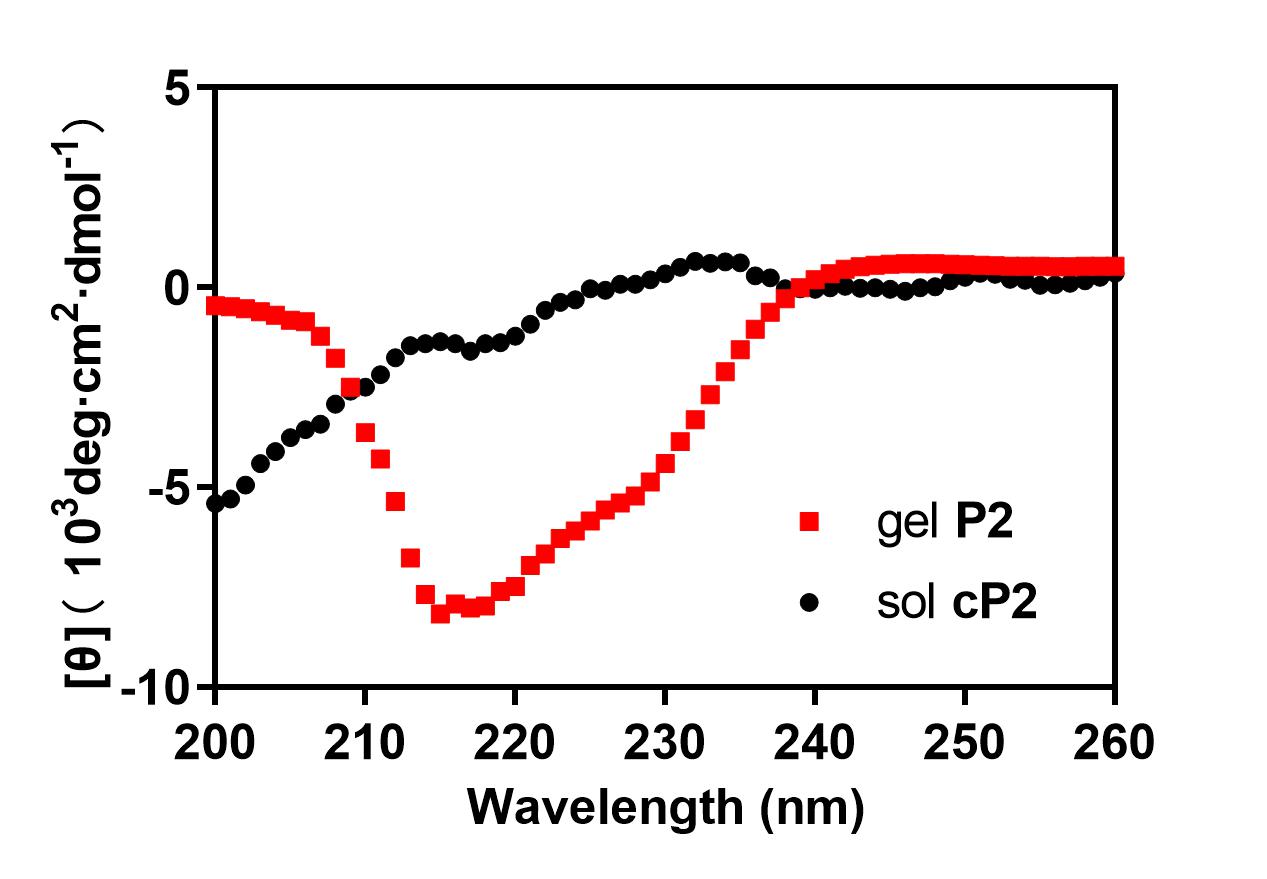


**Figure S7.** CD spectra of 2 wt% **P2** hydrogel and **cP2** solution.


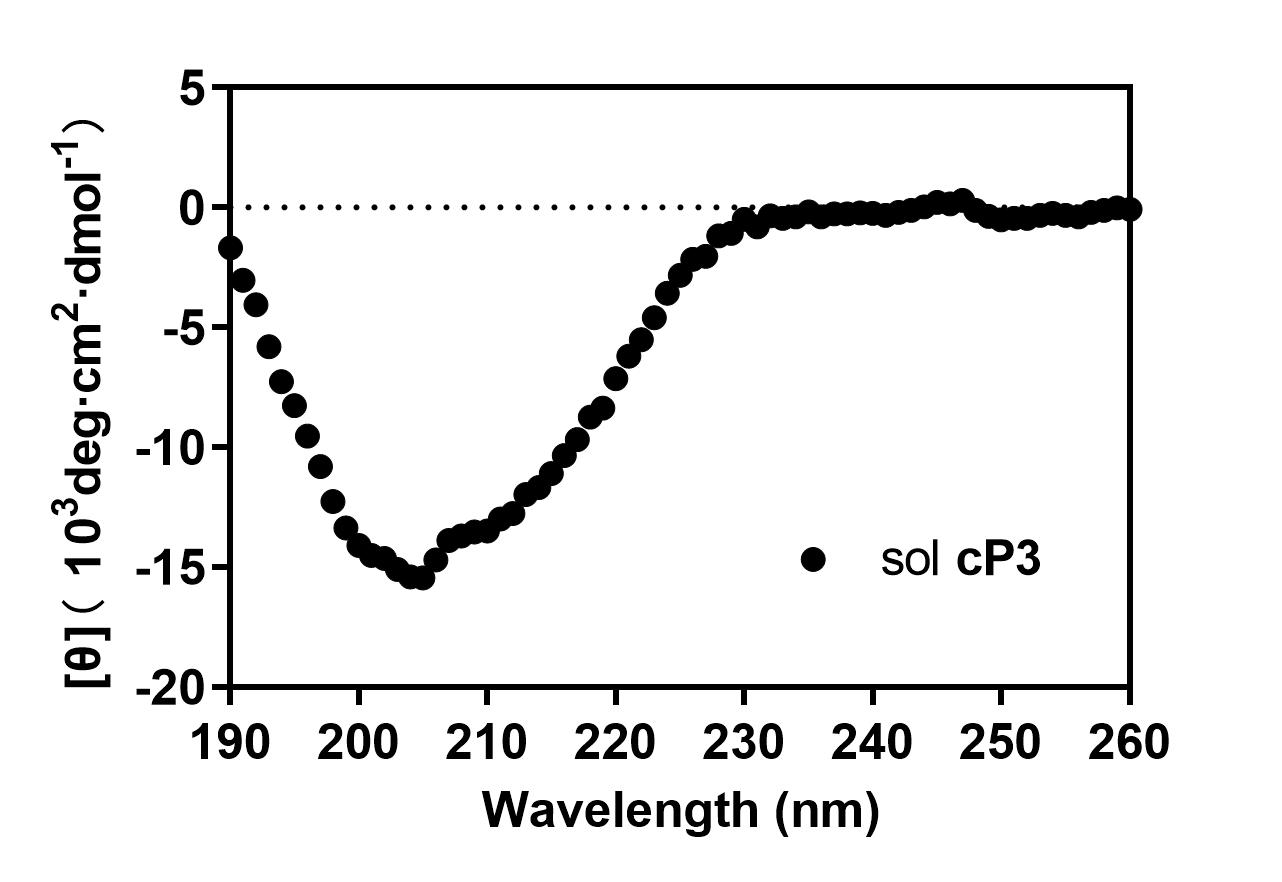


**Figure S8.** CD spectrum of 150 μM **cP3** in BTP buffer (10 mM BTP, 30 mM NaCl, pH 7.4).


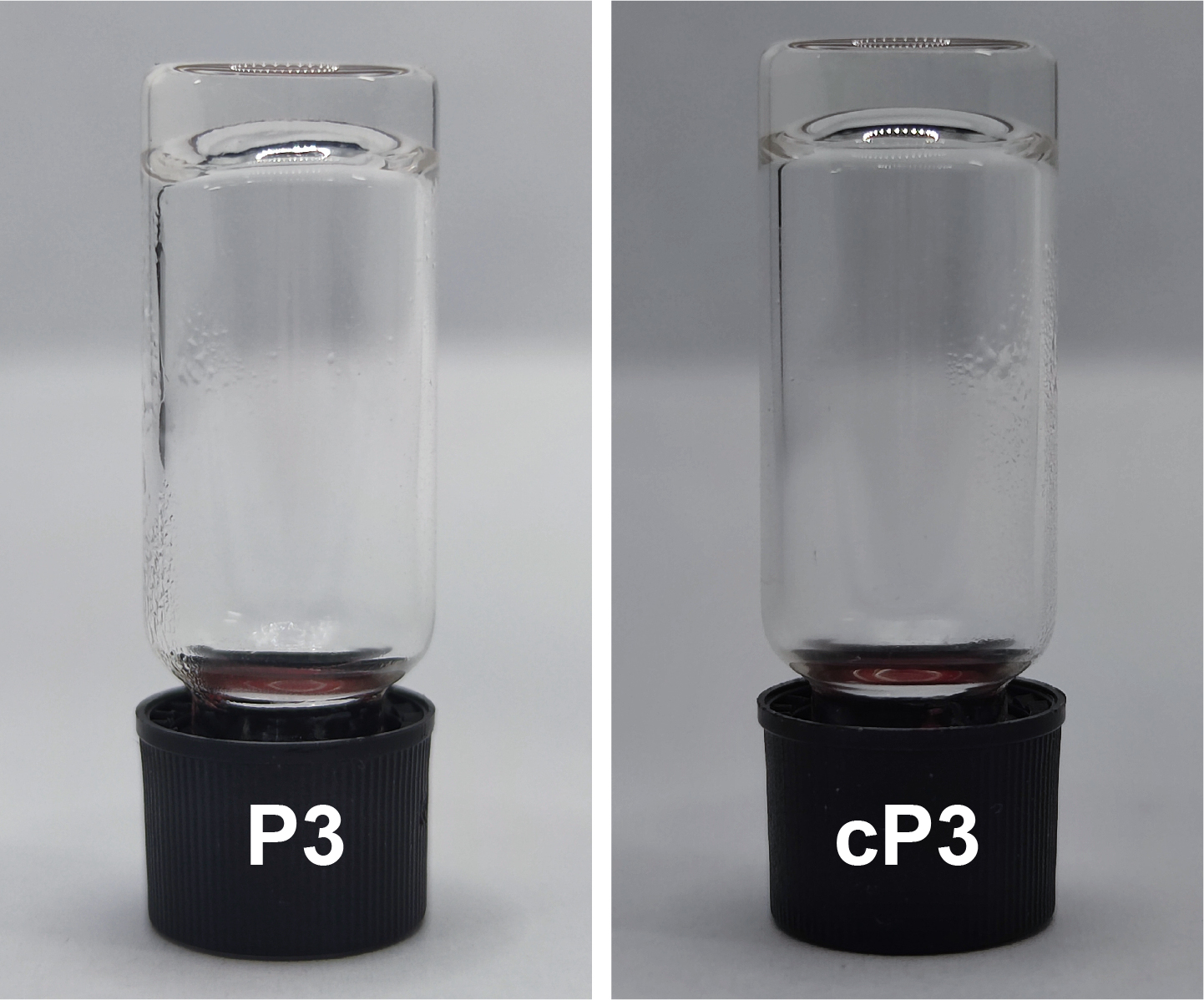


**Figure S9.** Optical images of **P3** hydrogel (left) and **cP3** hydrogel (right).


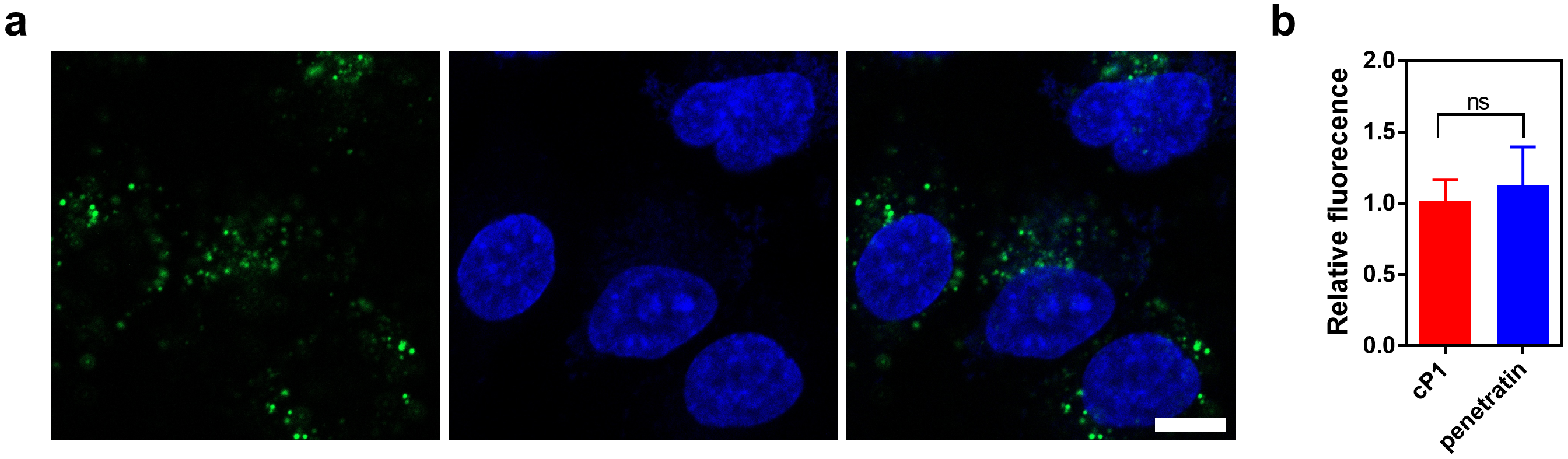
**Figure S10.** a) Confocal microscopy image of A549 cells after 4 h incubation with 5 μM fluorescently labeled penetratin. Scale bar: 10 μm. b) Quantification of mean fluorescence intensity of A549 cells after 4 h incubation with 5 μM **cP1** or penetratin, analyzed using ImageJ (n = 3). *P < 0.05, **P < 0.01, ***P < 0.001, and ns, not significance.


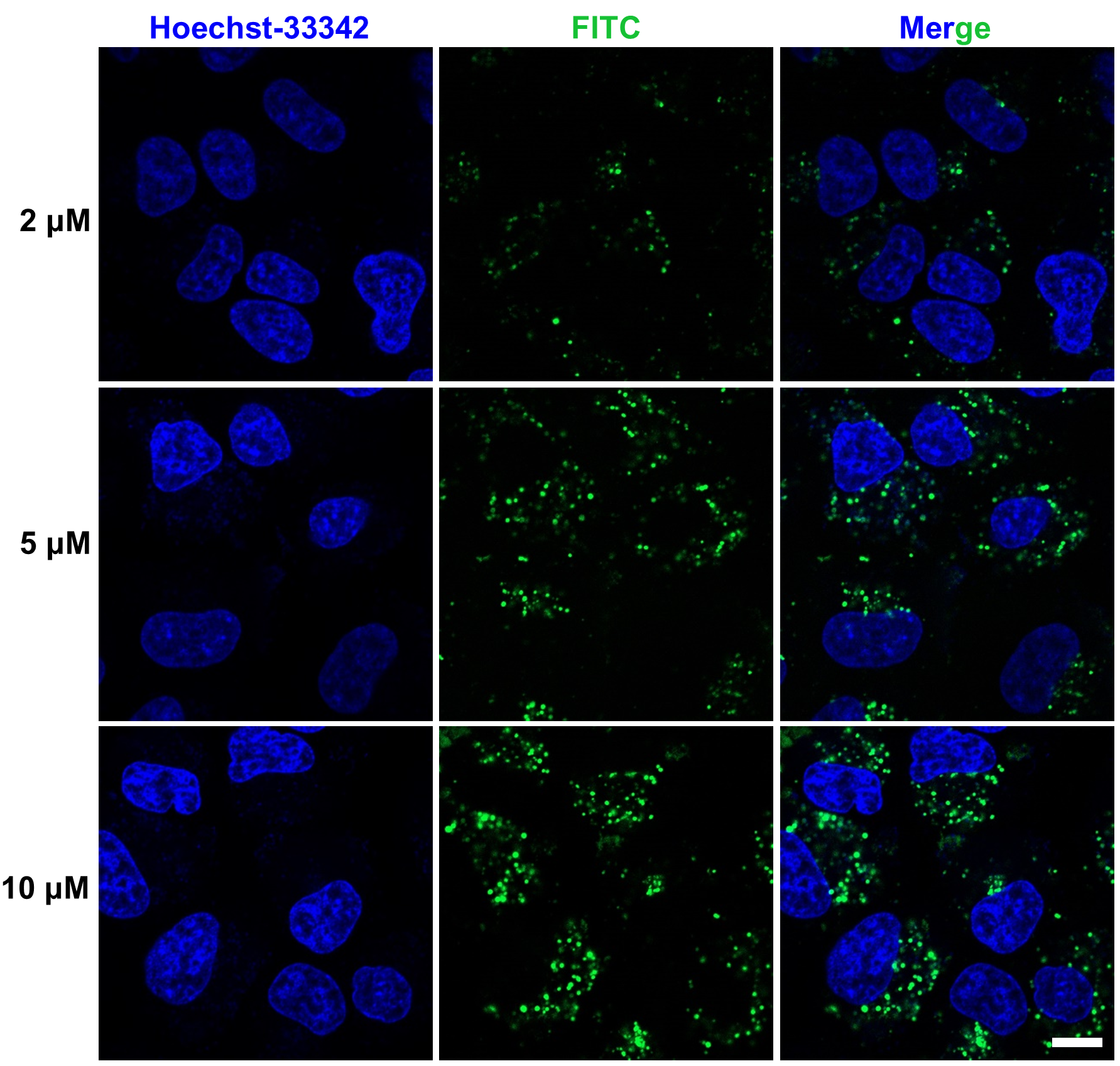


**Figure S11.** Confocal images showing concentration-dependent internalization of **FITC- cP1**. Scale bar: 10 μm.


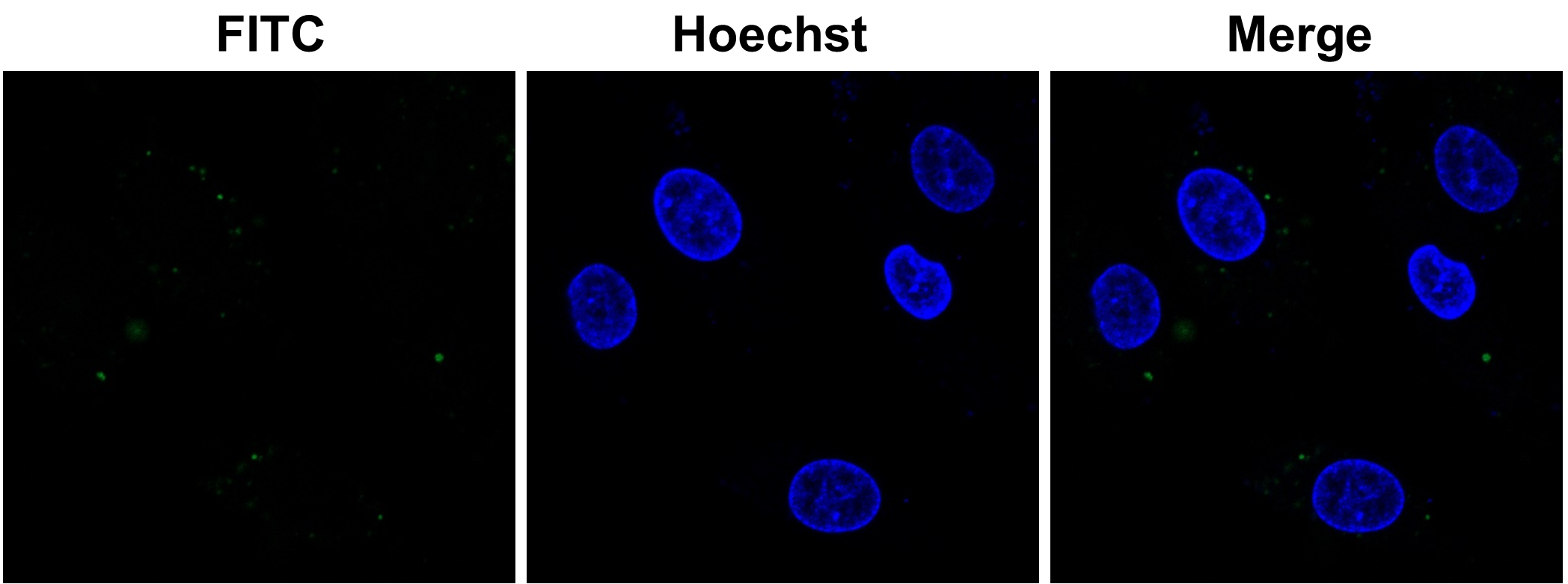


**Figure S12.** Confocal images of A549 cells after treatment with 5 μM **cP3** for 4 h. Scale bar: 10 μm.


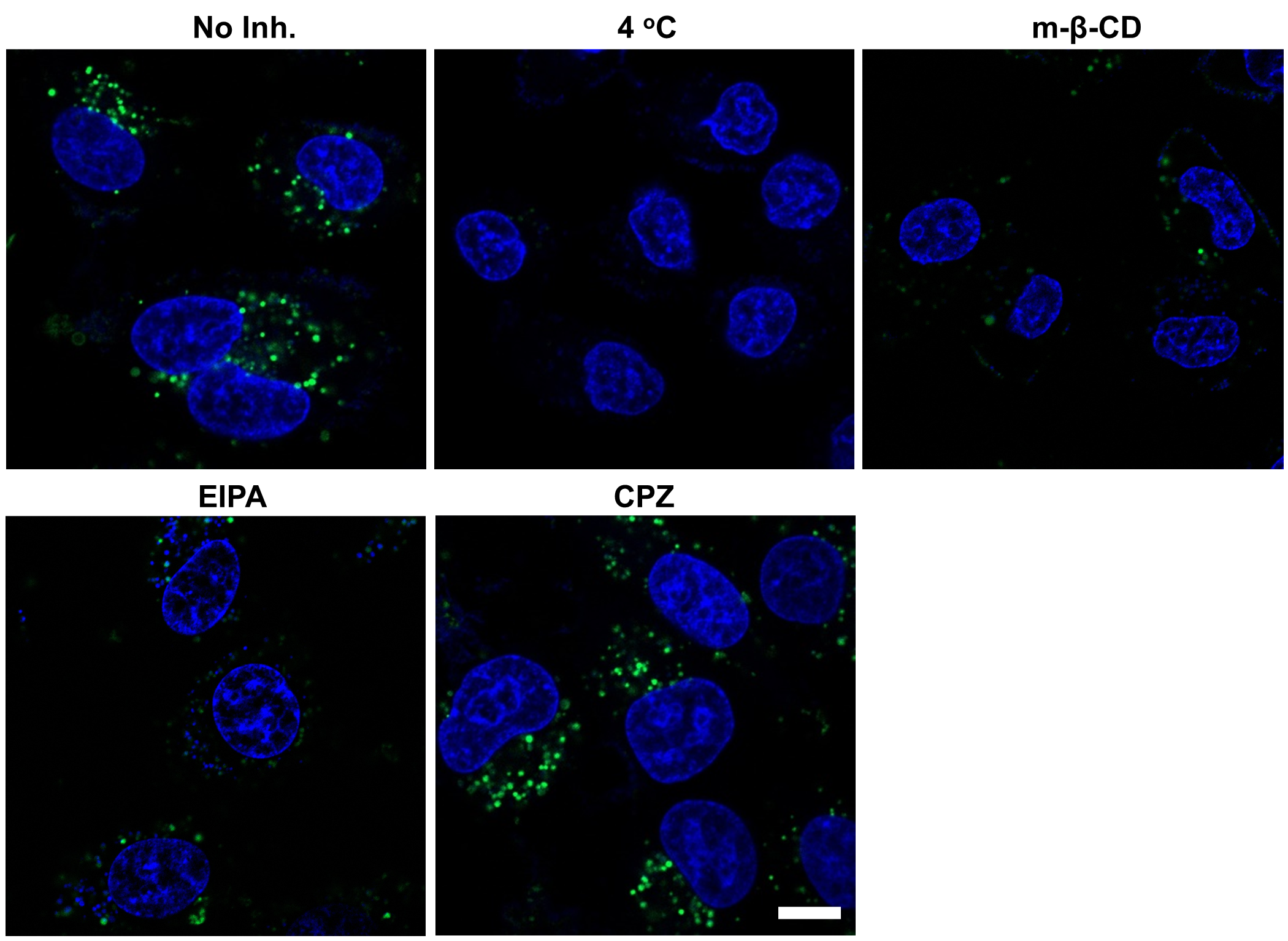


**Figure S13.** Confocal images showing uptake of 10 μM **FITC-cP1** in the presence of endocytosis inhibitors: CPZ (15 μM), M- b -CD (2.5 mM), and EIPA (30 μM), or at 4 °C. Scale bar: 10 μm.


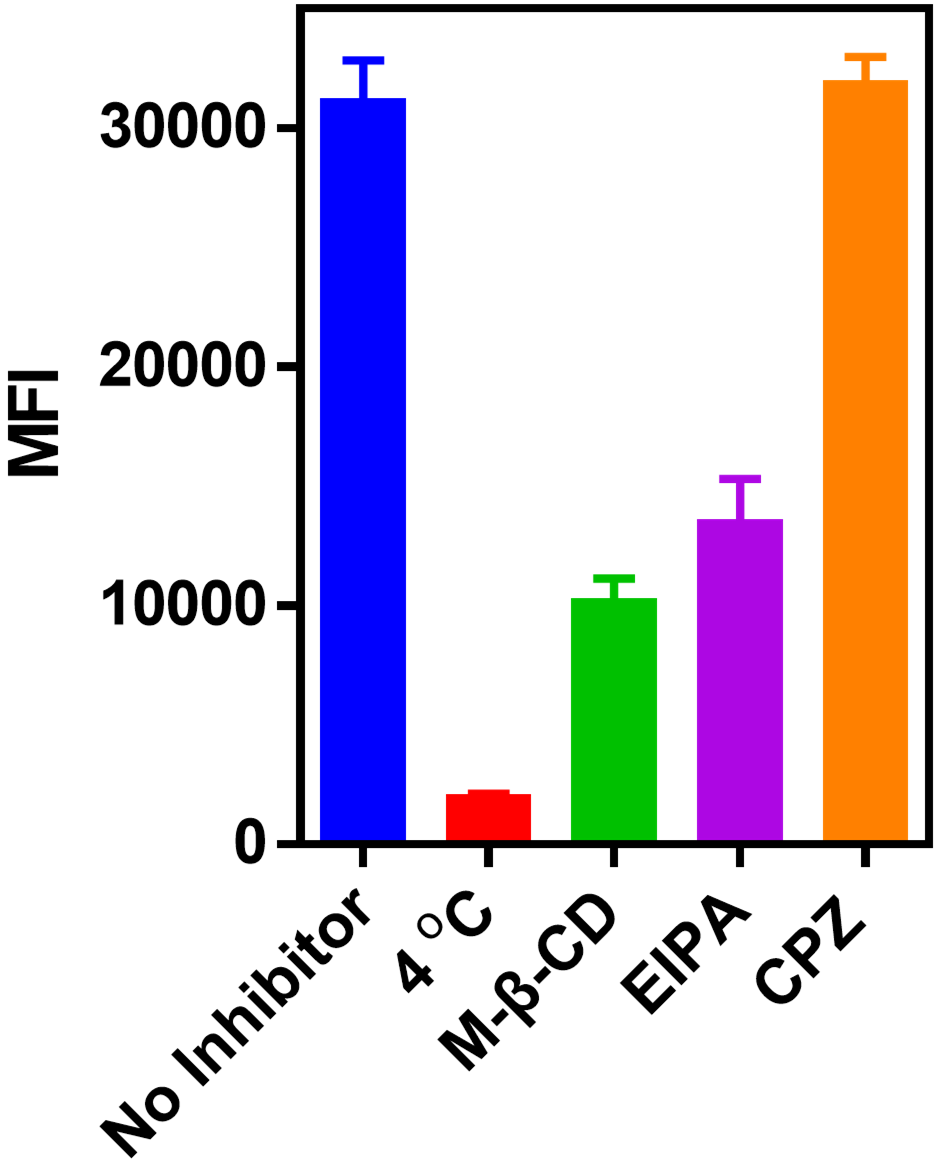


**Figure S14.** Quantification of **cP1** uptake using flow cytometry under the same conditions as **Figure S13**.


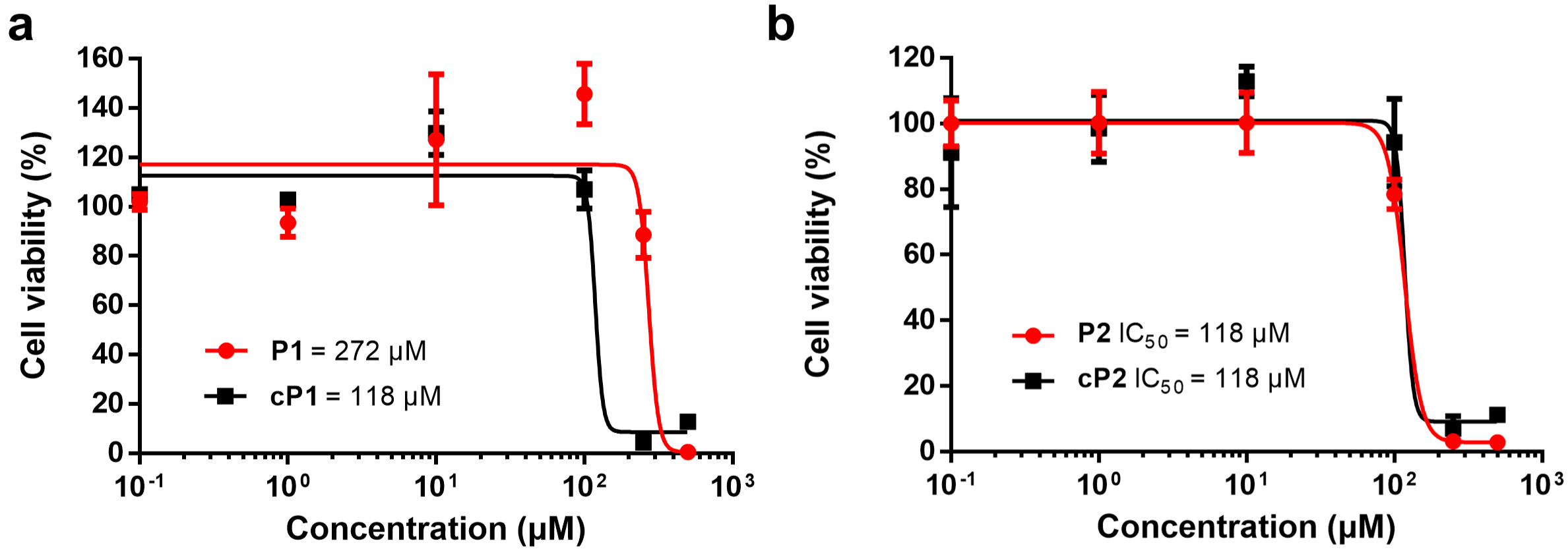


**Figure S15.** (a) Cytotoxicity of **P1** and **cP1** against A549 cells after 24 h (n =4). (b) Cytotoxicity of **P2** and **cP2** against SHED cells after 24 h (n =4).


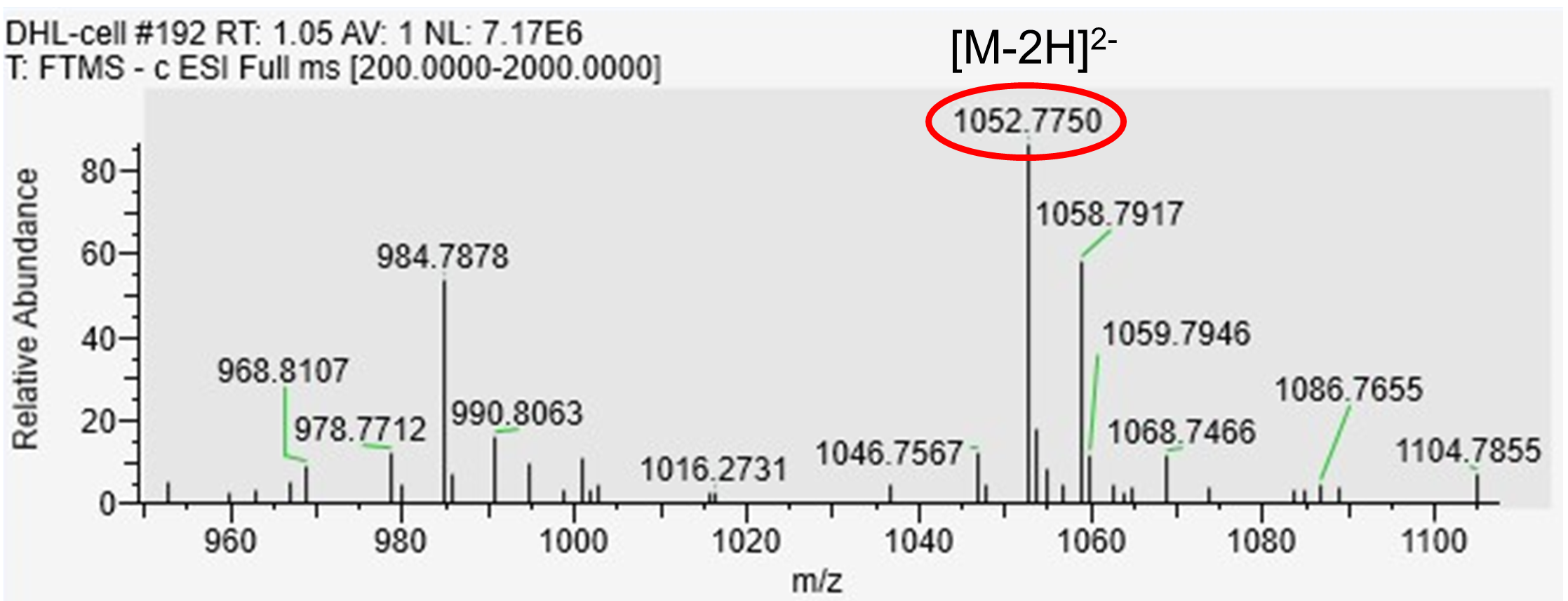


**Figure S16.** Mass spectrometry analysis of cell lysates from A549 cells treated with 10 μM **cP1** for 4 h.


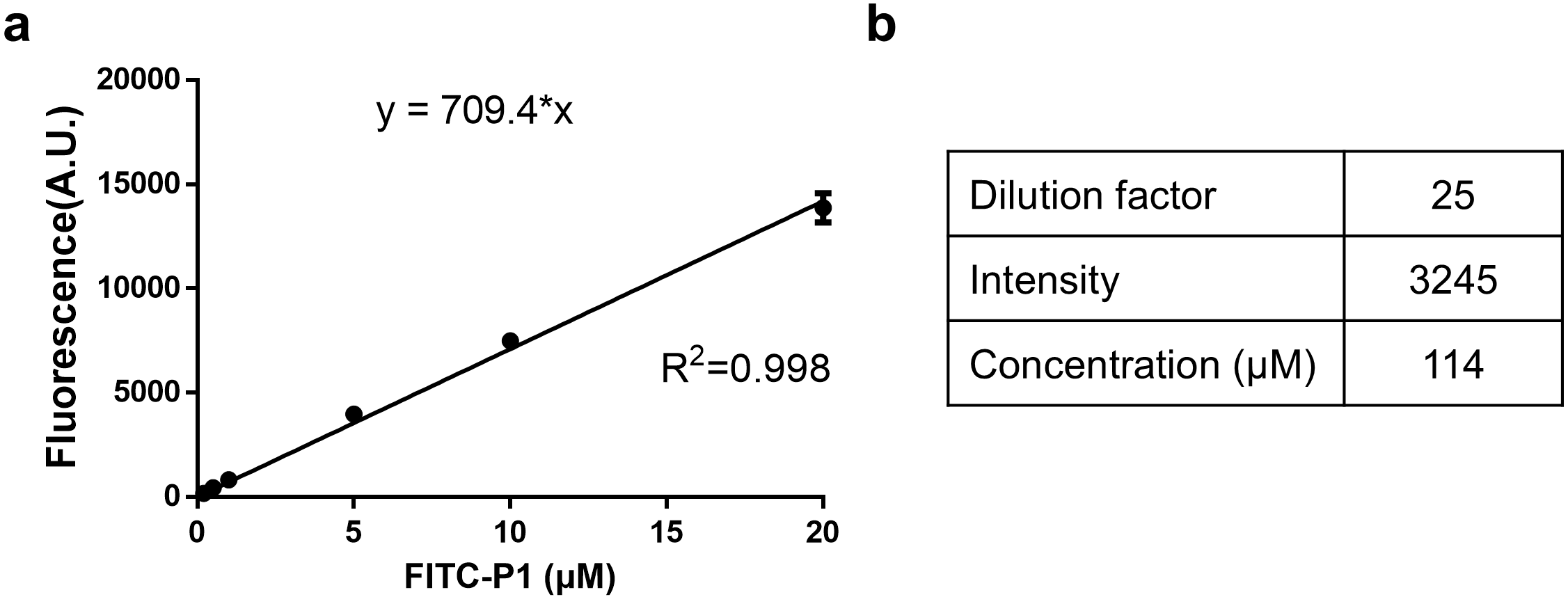


**Figure S17.** Fluorescence quantification of cell lysates. a) standard curve of fluorescence intensity vs. concentration of **FITC-P1**. b) Calculated intracellular concentration of **FITC-P1** (n = 3).


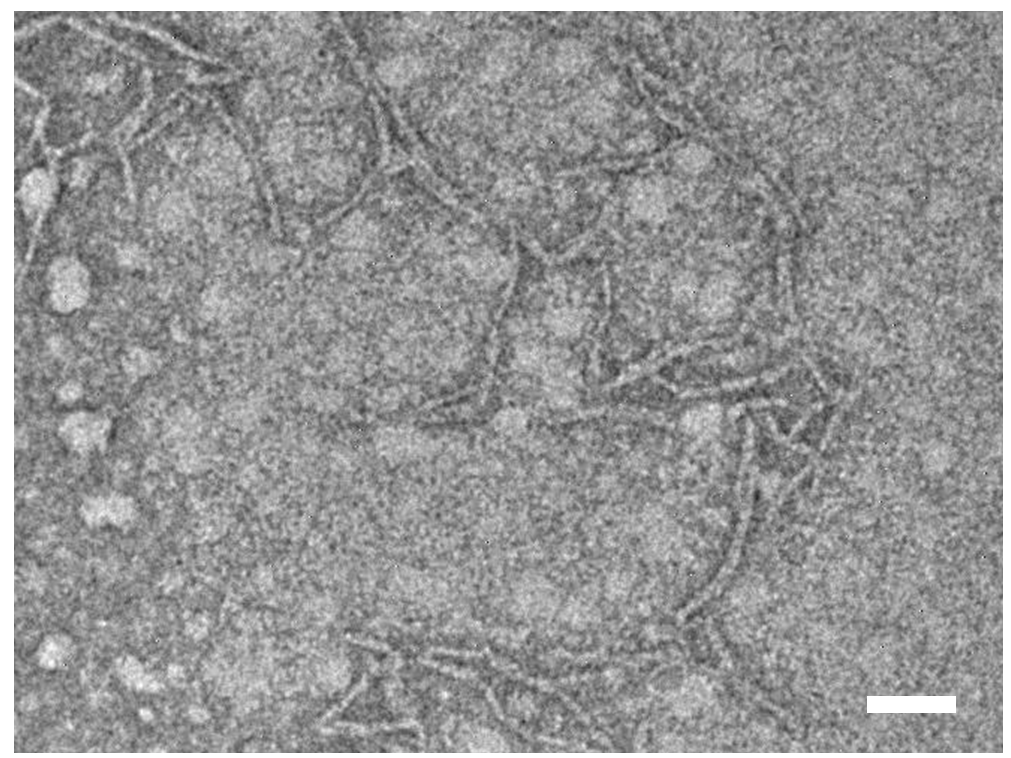


**Figure S18.** TEM image of 100 μM **P1** in BTP buffer. Scale bar: 50 nm.


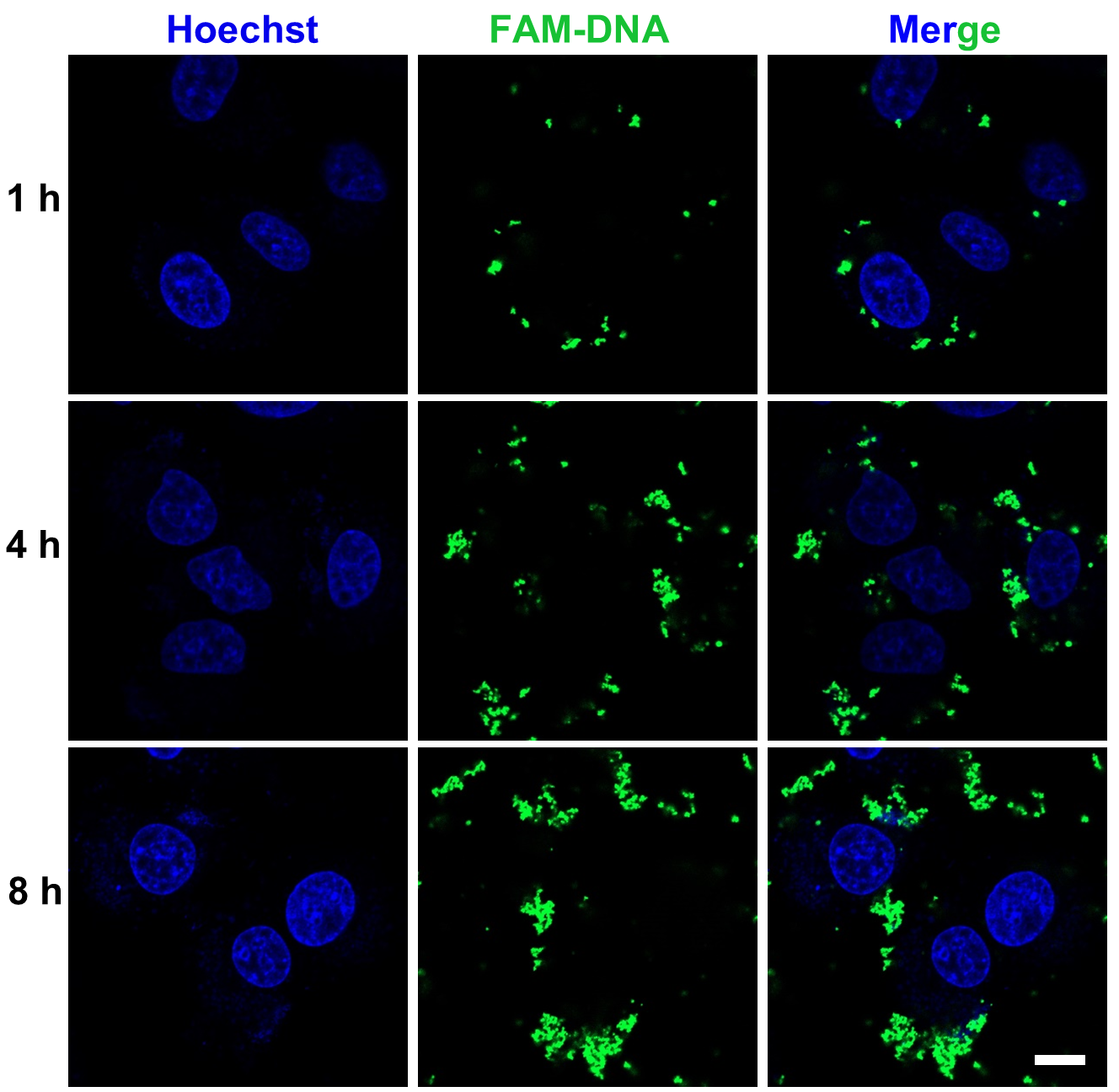


**Figure S19.** Confocal images showing time-dependent cellular internalization of **cP1** and FAM-DNA. Scale bar: 10 μm.


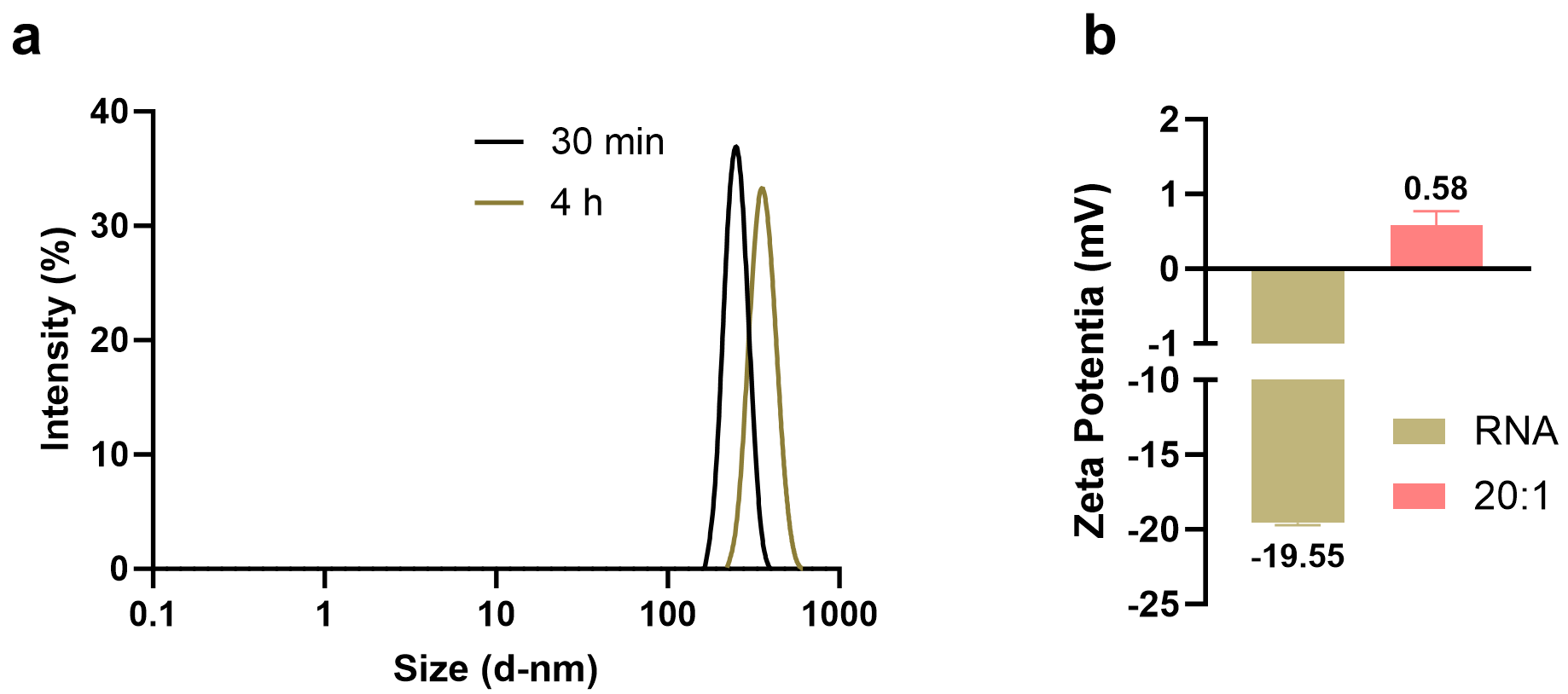


**Figure S20.** DLS (a) and zeta-potential (b) characterization of **cP1**/miRNA complexes.


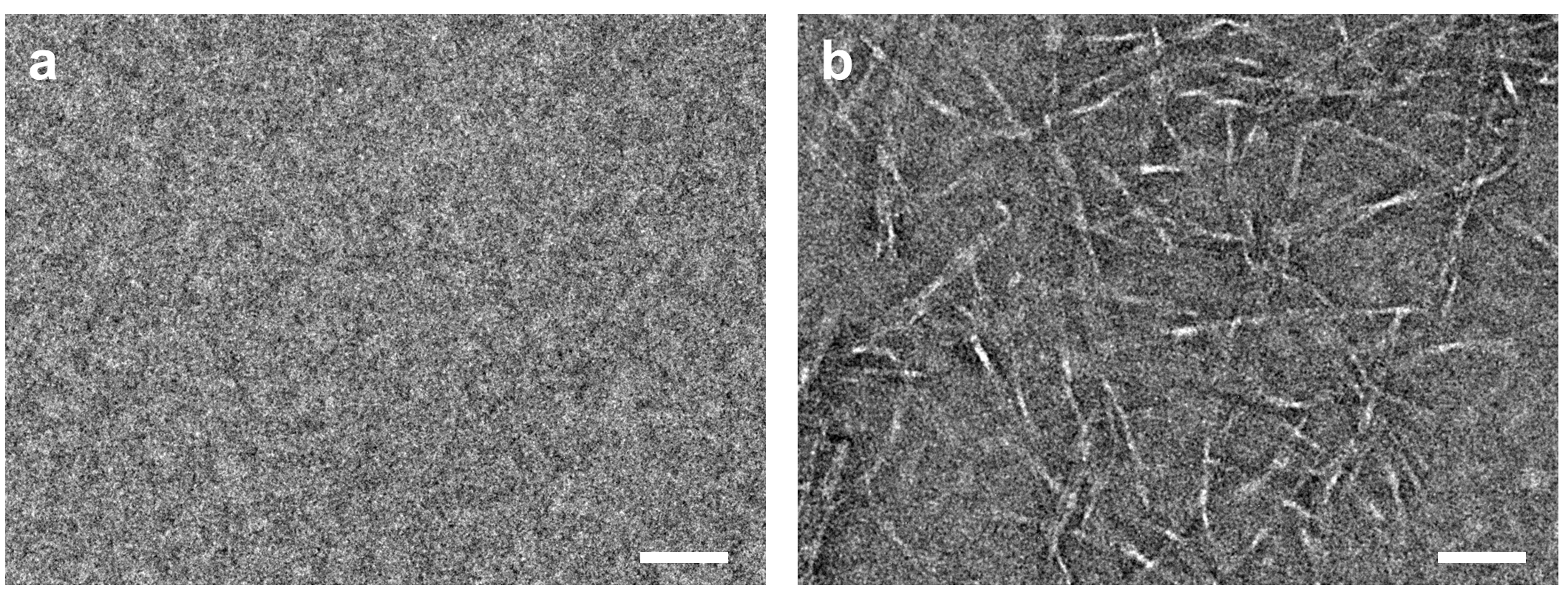


**Figure S21.** a) TEM image of 1 mM **cP1** in BTP buffer. b) TEM image of 1 mM **cP1** solution treated with TCEP for 10 min. Scale bar: 50 nm.


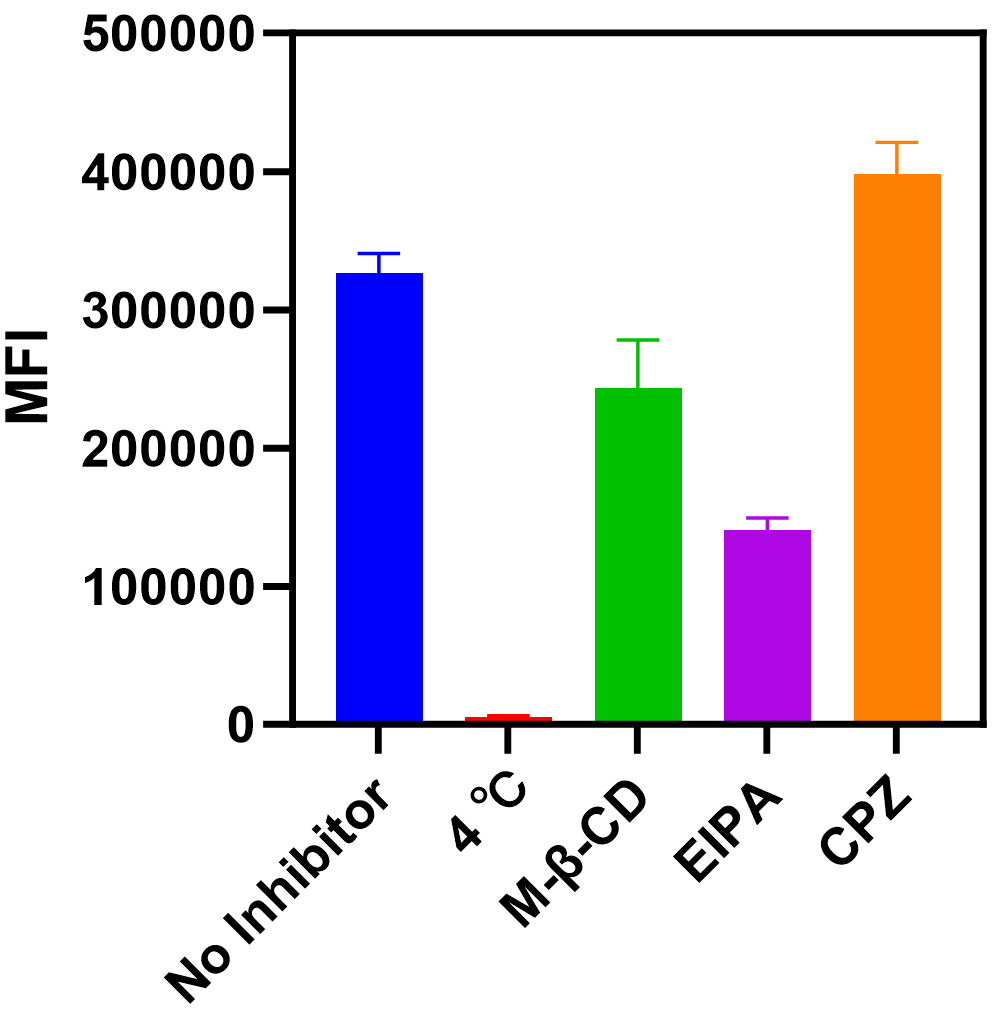


**Figure S22.** Quantification of **cP1**/miRNA complexes uptake by A549 cells using flow cytometry.


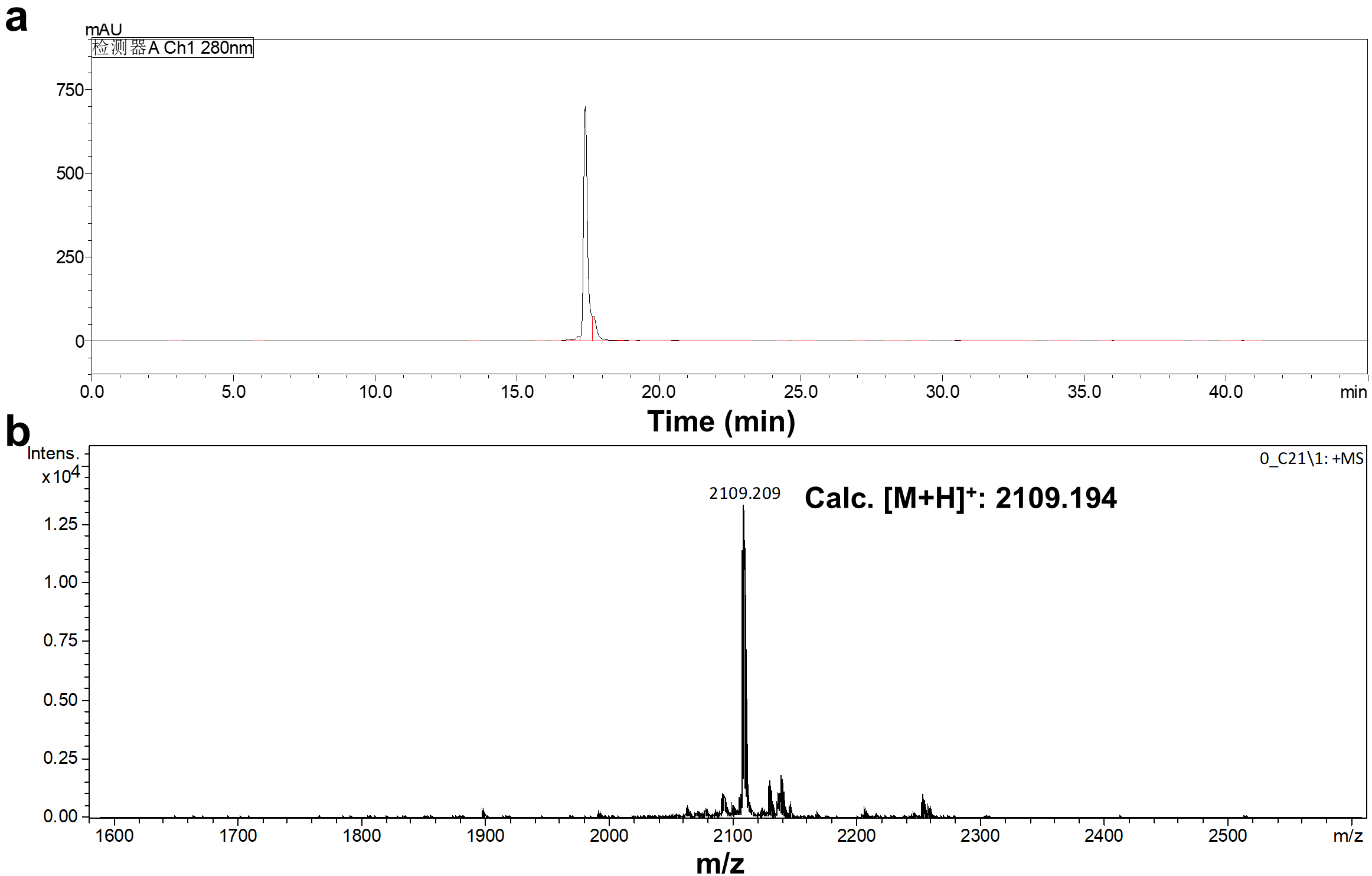


**Figure S23.** a) Analytical HPLC chromatogram and b) MALDI-TOF MS spectrum of peptide **P1**.


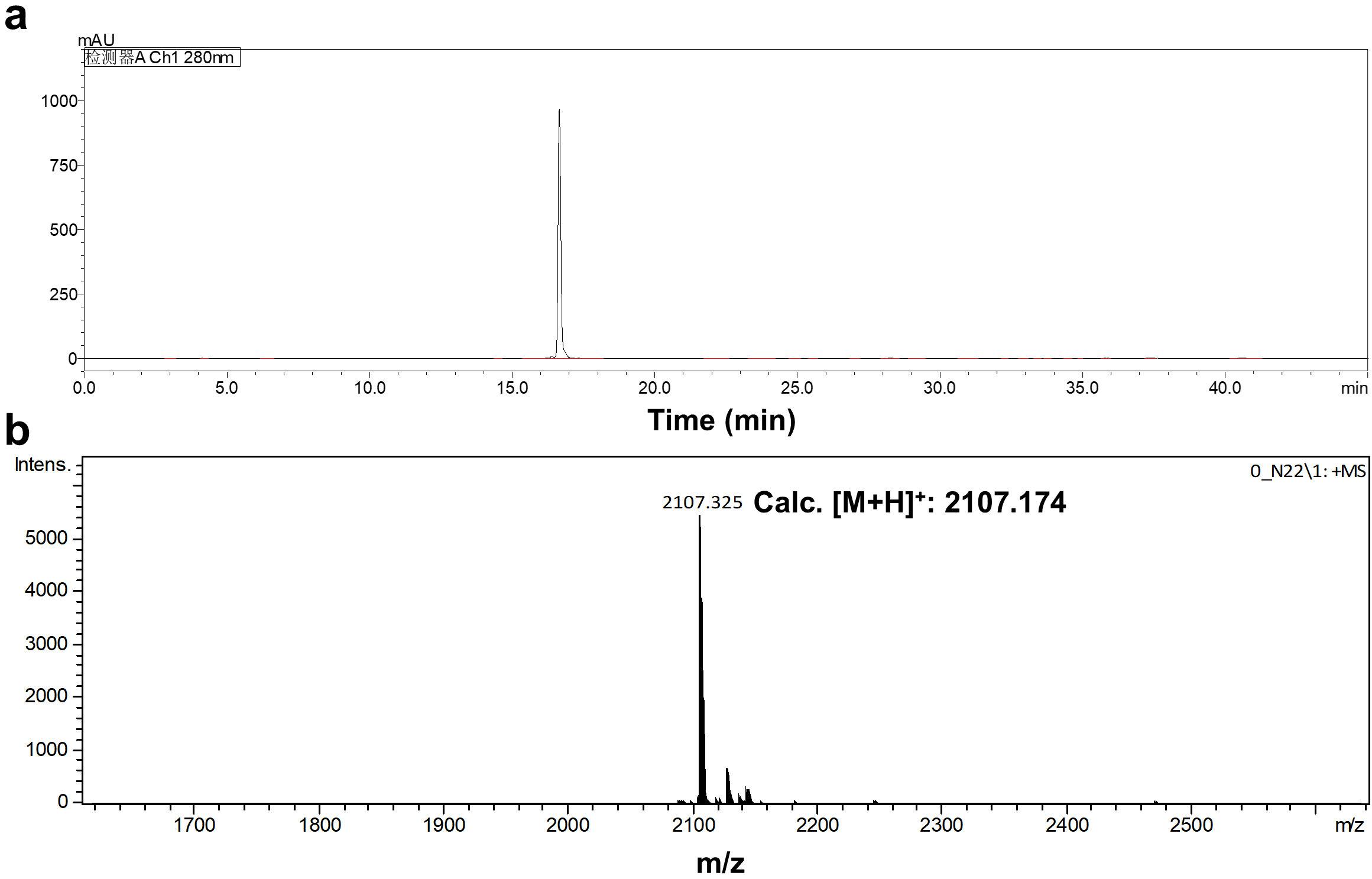


**Figure S24.** a) Analytical HPLC chromatogram and b) MALDI-TOF MS spectrum of peptide **cP1**.


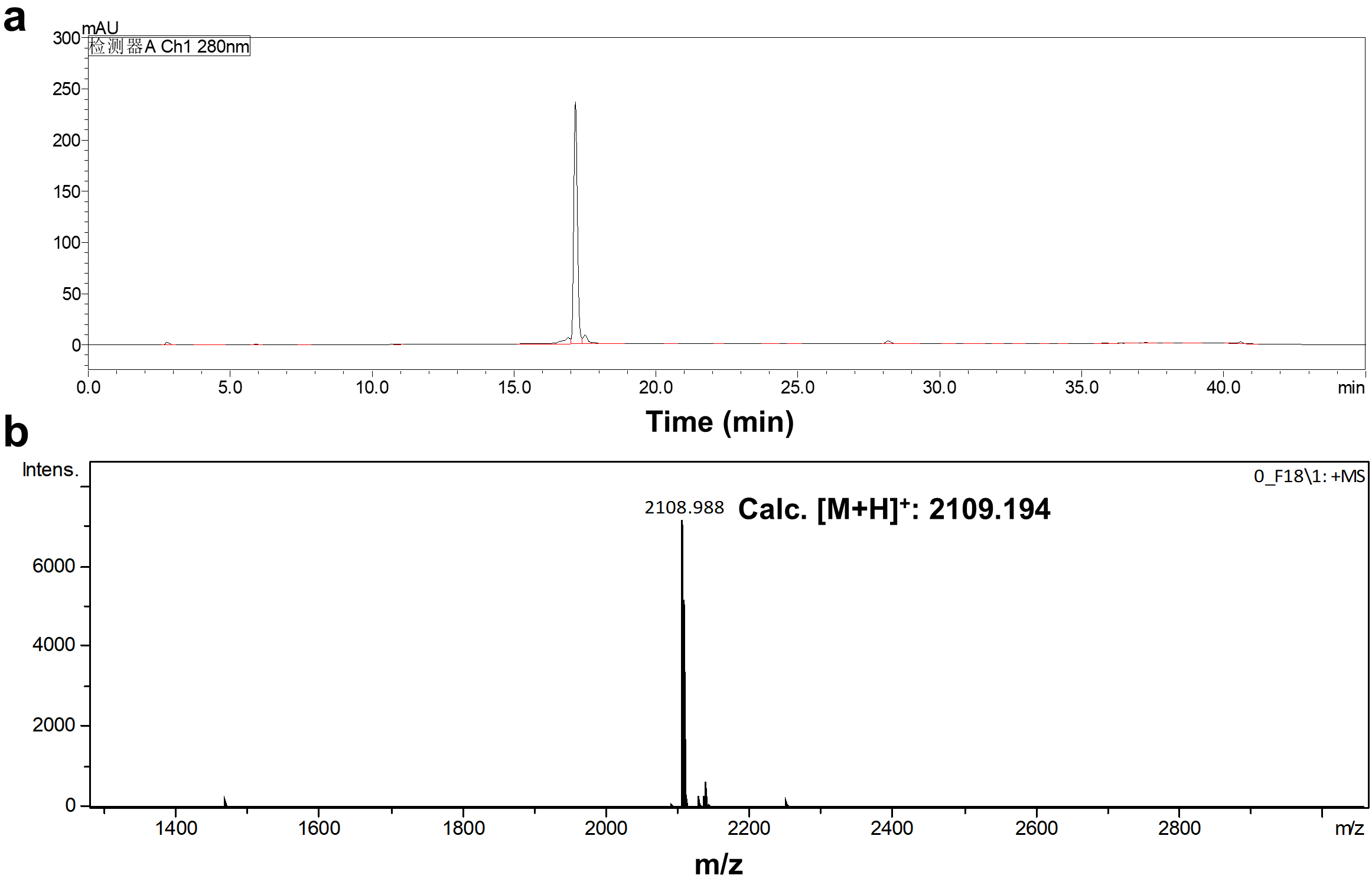


**Figure S25.** a) Analytical HPLC chromatogram and b) MALDI-TOF MS spectrum of peptide **P2**.


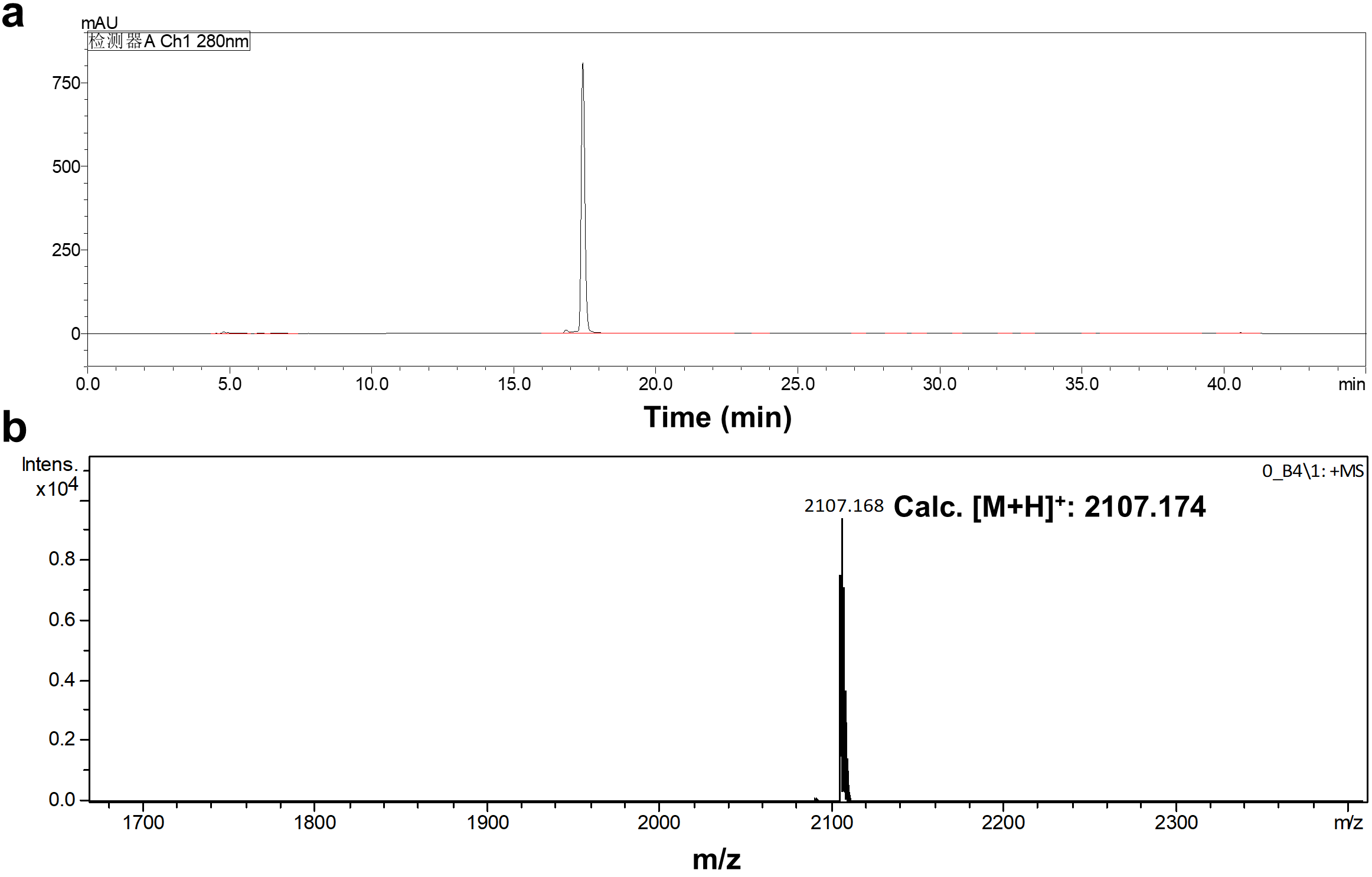


**Figure S26.** a) Analytical HPLC chromatogram and b) MALDI-TOF MS spectrum of peptide **cP2**.


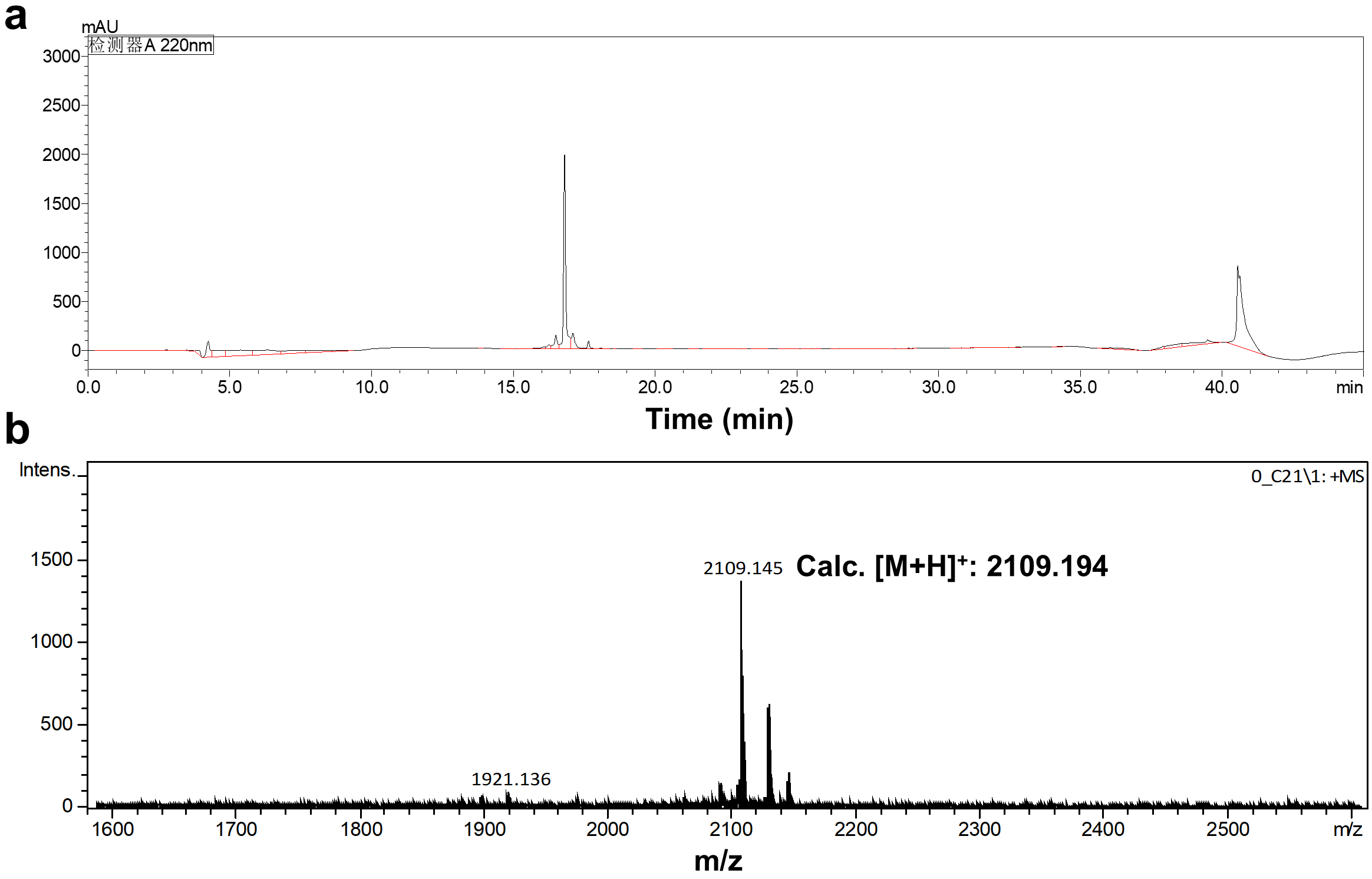


**Figure S27.** a) Analytical HPLC chromatogram and b) MALDI-TOF MS spectrum of peptide **P3**.


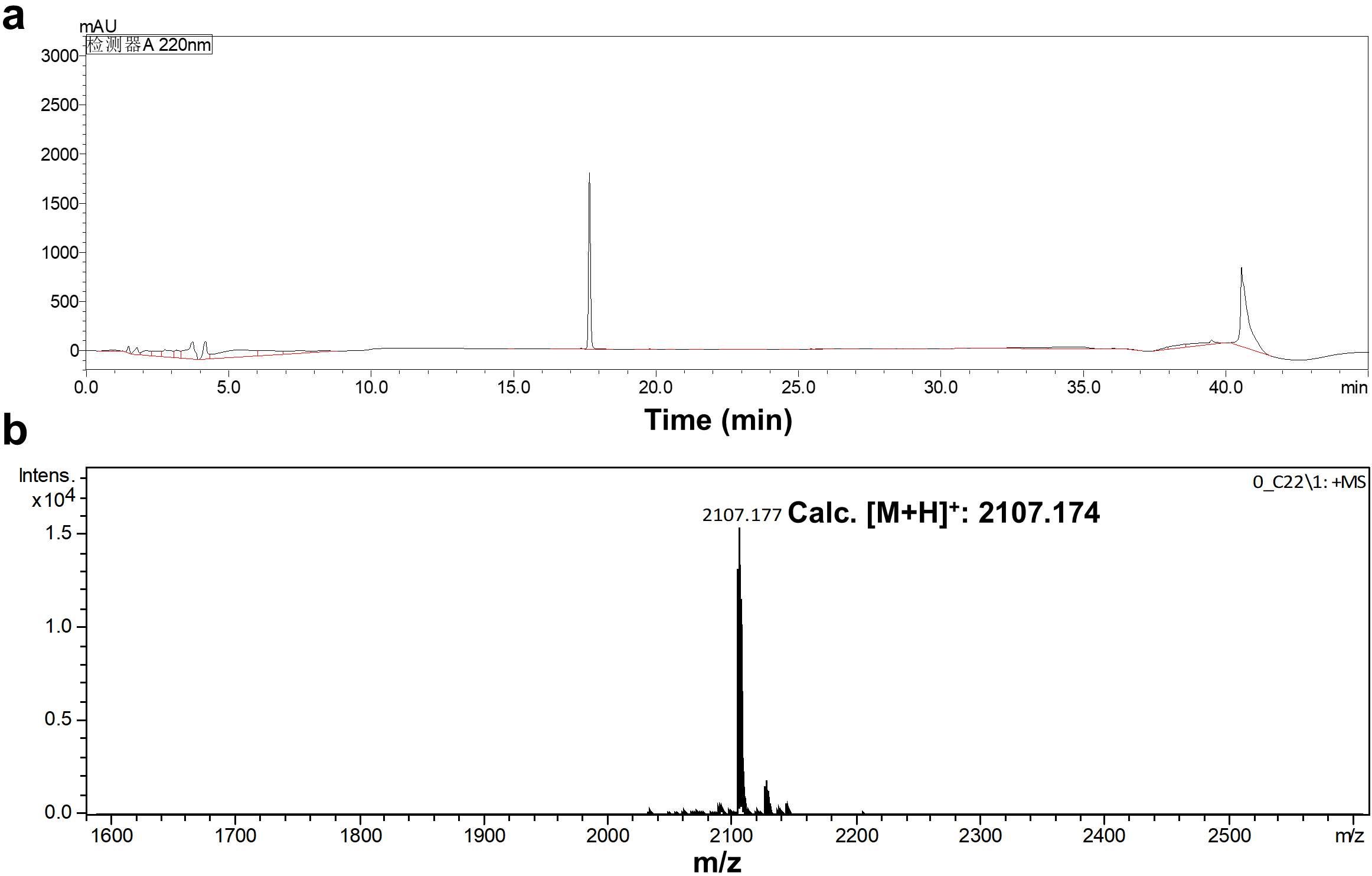


**Figure S28.** a) Analytical HPLC chromatogram and b) MALDI-TOF MS spectrum of peptide **cP3**.


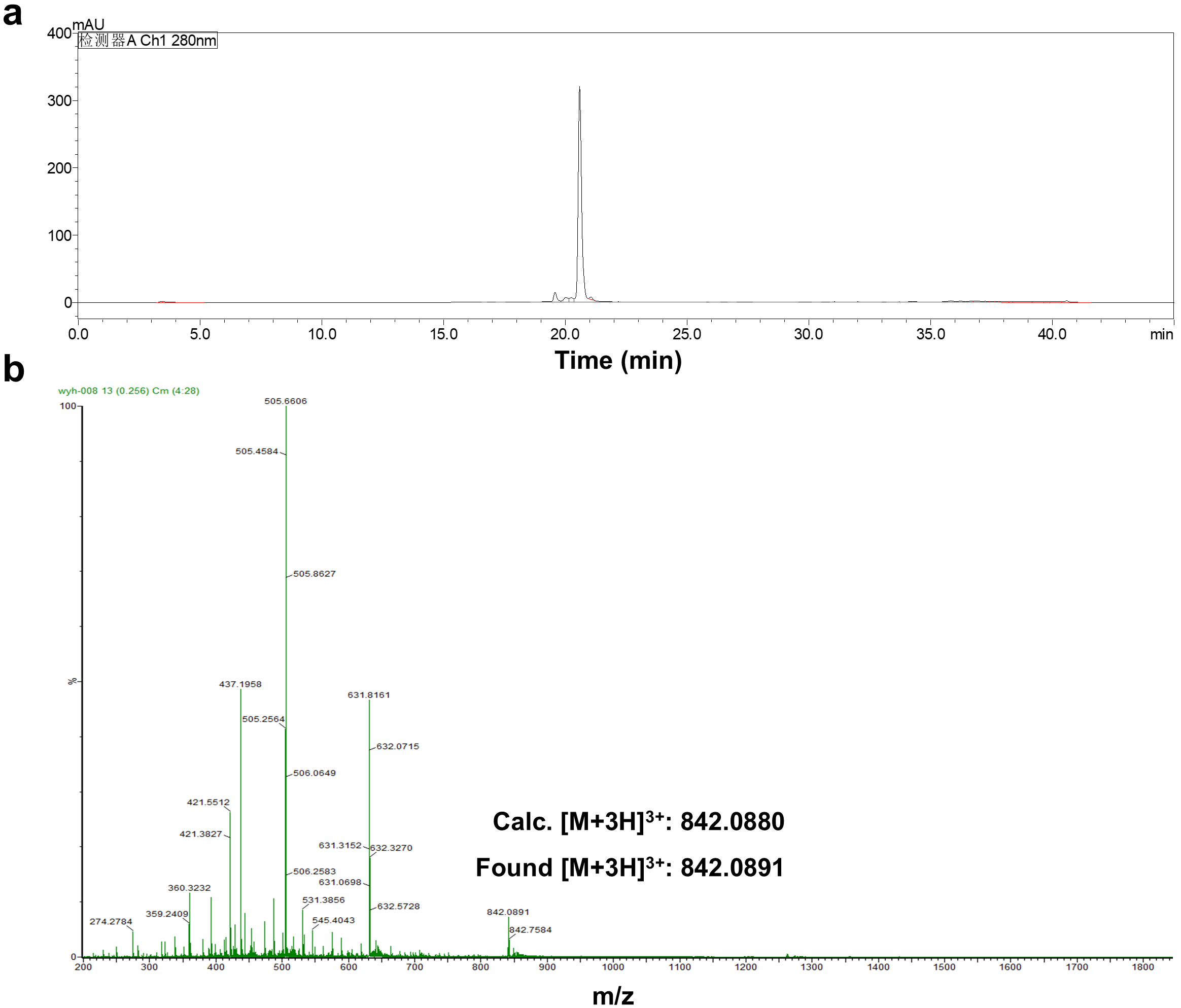


**Figure S29.** a) Analytic HPLC chromatogram and b) MS spectrum of peptide **FITC-P1**.


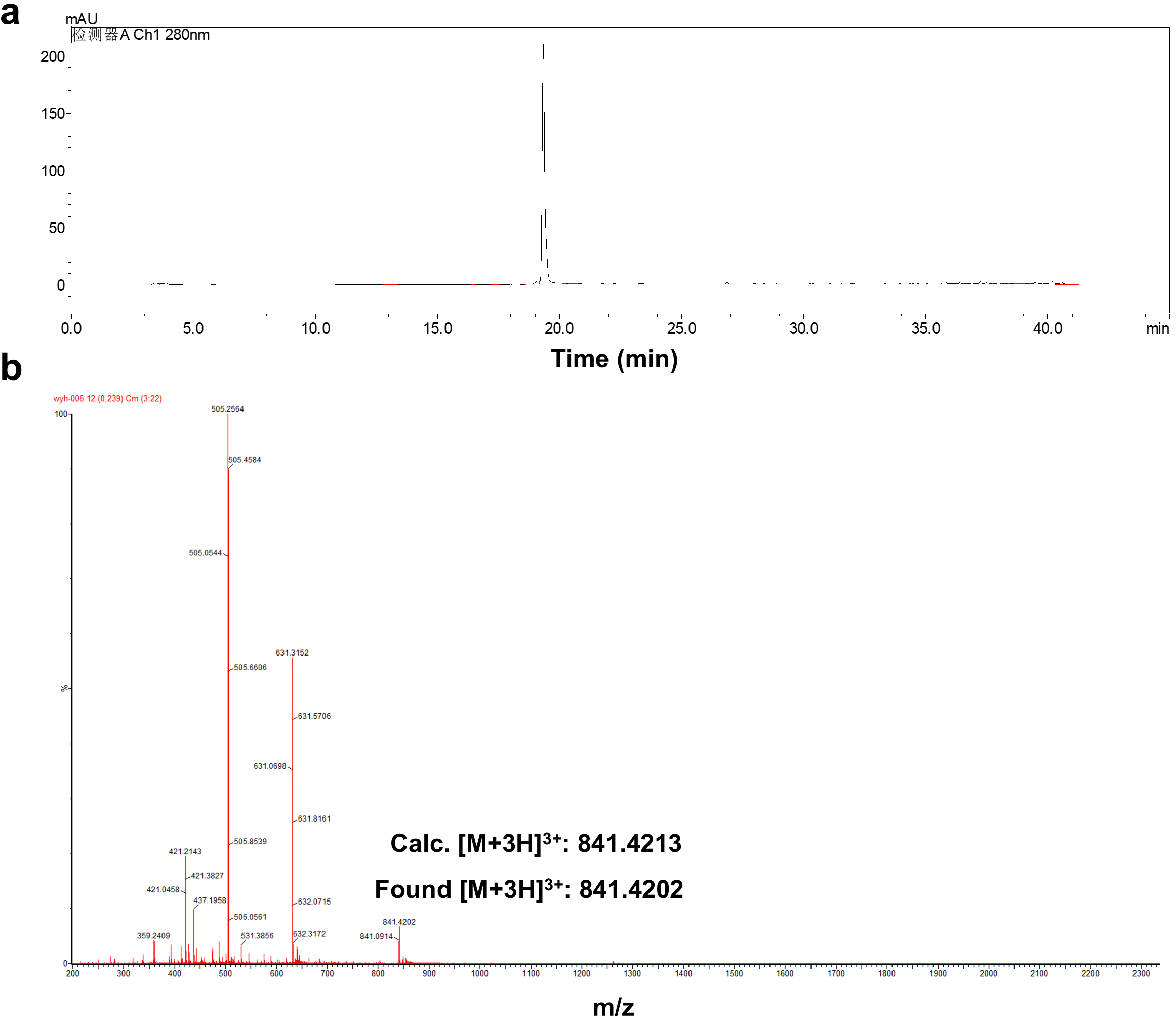


**Figure S30.** a) Analytic HPLC chromatogram and b) MS spectrum of peptide **FITC-cP1**.

**
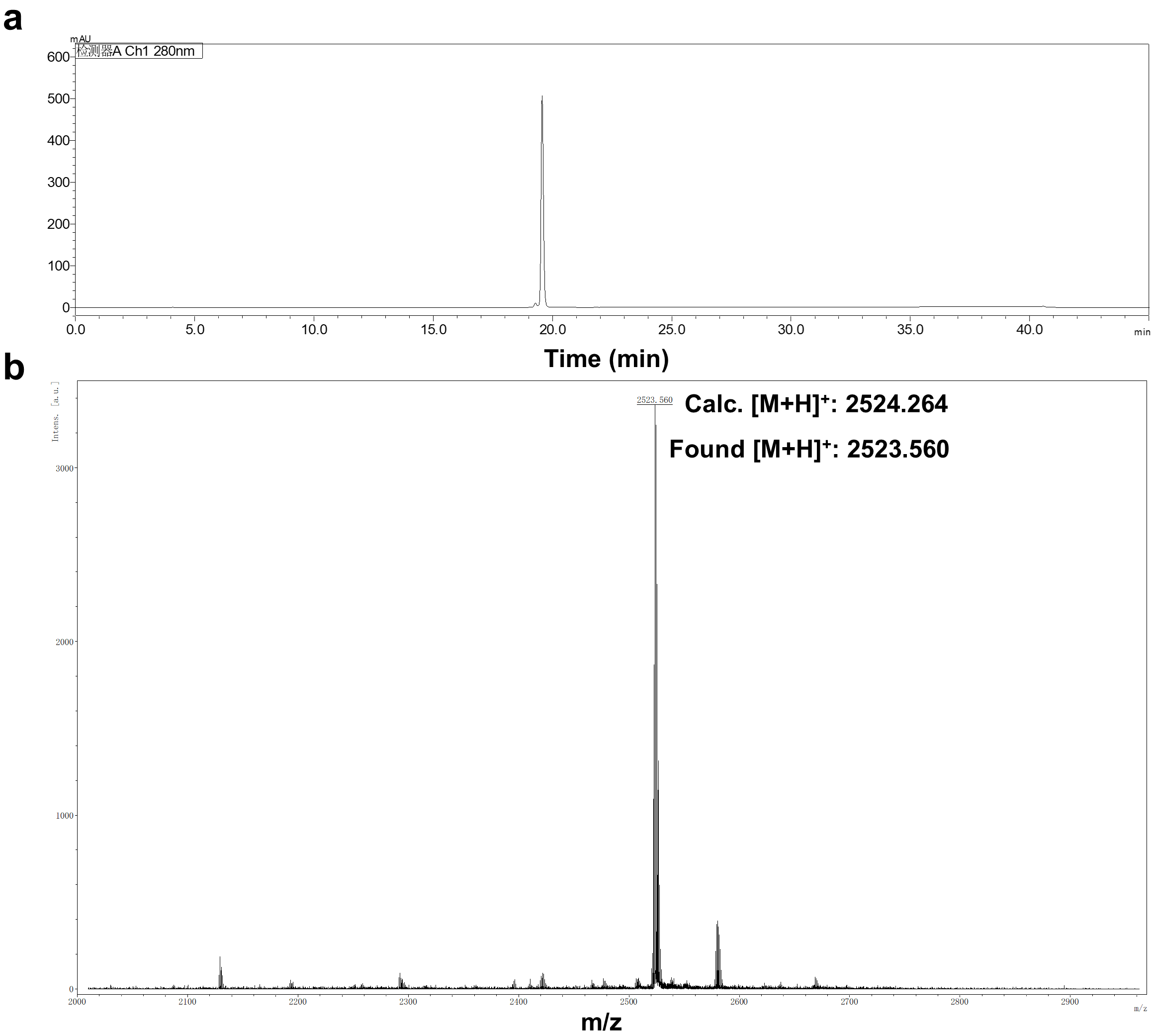
**

**Figure S31.** a) Analytic HPLC chromatogram and b) MS spectrum of peptide **FITC-P3**.


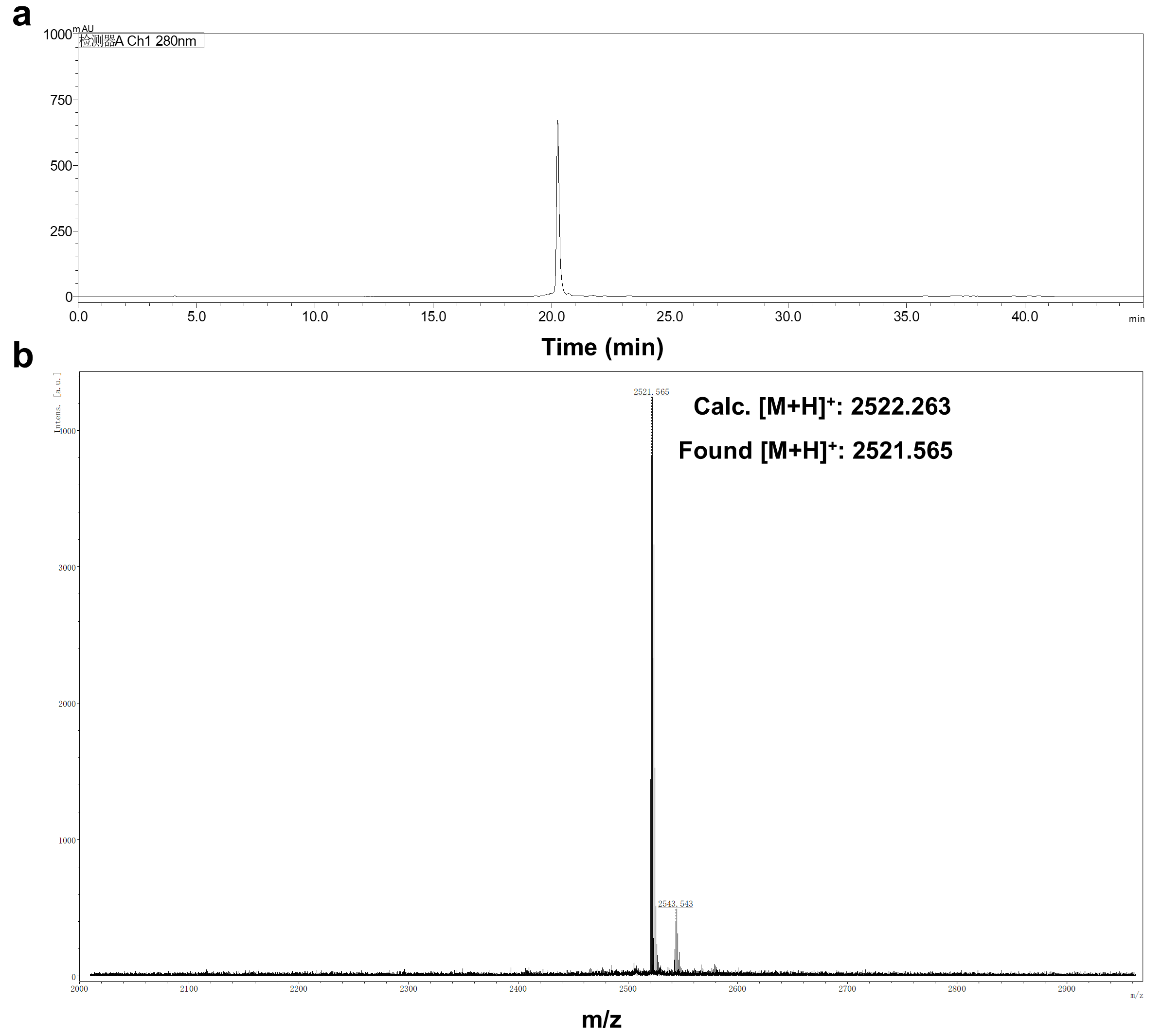


**Figure S32.** a) Analytic HPLC chromatogram and b) MS spectrum of peptide **FITC-cP3**.
